# Supplementary material for: Accounting for technical noise in differential expression analysis of single-cell RNA sequencing data
Source: Nucleic Acids Res. 2017 Sep 25;45(19):10978–88. doi: 10.1093/nar/gkx754 (PMC5737676; doi:10.1093/nar/gkx754)
Supplement: Supplementary Data [file gkx754_supp.pdf]

Accounting for technical batch effects in single-cell RNA sequencing analysis

Supplemental Methods

February 17, 2017

Contents

1 Generative model of single-cell RNA sequencing **2**

1.1 Modelling spike-ins . . . 2
1.2 Empirical Bayes estimation of cell-specific technical parameters . . . 3
1.3 Modelling biological genes . . . 9
1.4 Differential expression analysis . . . 11
1.5 Expectation-Maximization algorithm . . . 12
1.6 Estimation of cell size factor . . . 13

2 Evaluation of Performance and Comparison with Other Methods **14**

2.1 Type I error rates . . . 14
2.2 Impact of batch effects . . . 14
2.3 Power . . . 15
2.3.1 Power and effect size . . . 18
2.3.2 Power and sample size . . . 28
2.4 Application to real data . . . 30
2.4.1 Zeisel et al. data . . . 30
2.4.2 SCAP-T data . . . 31

3 Computational Details **43**

3.1 Laplace Approximation . . . 43
3.2 Session info for running DESeq2, MAST, SCDE . . . 44
3.3 Session info for running SCRAN . . . 46
3.4 Code snippets for running DESeq2, MAST, SCDE and SCRAN . . . 46

1 Generative model of single-cell RNA sequencing

1.1 Modelling spike-ins

Let Y_{cg} be the observed number of reads or transcripts (if UMI is used) for the spiked-in molecule g in cell c. Let μ_g be the true number of molecules of g added to each cell lysate. Given the cell-specific technical parameters $(\alpha_c, \beta_c, \kappa_c, \tau_c)$, the distribution of Y_{cg} can be modelled with the following steps:

Step.*a* Let Z_{cg} be the indicator that dropout does not occur, i.e. the gene is captured in the library. The probability of $Z_{cg} = 1$ (π_{cg}) depends on the amount of added spike-in g, μ_g. A logistic model can be used to describe this relationship.

$$\begin{aligned} \pi_{cg} &= \text{expit}\left[\kappa_c + \tau_c \log \mu_g\right] \\ Z_{cg} &\sim \text{Bernoulli}\left(\pi_{cg}\right) \end{aligned} \tag{1}$$

Step.*b* Let λ_{cg} be the expected value for the read count of spike-in g in cell c.

$$\log \lambda_{cg} = \alpha_c + \beta_c \log \mu_g. \tag{2}$$

Step.*c* Given the status of Z_{cg}, the observed count for spike-in g in cell c Y_{cg} can be modelled as,

$$Y_{cg} | Z_{cg} \sim \begin{cases} \text{Poisson}(\lambda_{cg}), & \text{if } Z_{cg} = 1 \\ 0, & \text{if } Z_{cg} = 0 \end{cases}. \tag{3}$$

The conditional probability density function of Y_{cg} given Z_{cg} is,

$$\Pr\left[Y_{cg} | Z_{cg} = 0\right] = \begin{cases} 1, & \text{if } Y_{cg} = 0 \\ 0, & \text{if } Y_{cg} > 0 \end{cases} \tag{4}$$

$$\Pr\left[Y_{cg} | Z_{cg} = 1\right] = \frac{\lambda_{cg}^{Y_{cg}} e^{-\lambda_{cg}}}{y_{cg}!}. \tag{5}$$

Step.*d* We can arrive at the marginal likelihood of Y_{cg} by summing over the support of Z_{cg},

$$\begin{aligned} \Pr\left[Y_{cg}\right] &= \sum_{Z_{cg}} \Pr\left[Y_{cg}, Z_{cg}\right] \\ &= \sum_{Z_{cg}} \Pr\left[Y_{cg} | Z_{cg}\right] \Pr\left[Z_{cg}\right] \\ &= \Pr\left[Y_{cg} | Z_{cg} = 0\right] \Pr\left[Z_{cg} = 0\right] + \Pr\left[Y_{cg} | Z_{cg} = 1\right] \Pr\left[Z_{cg} = 1\right] \\ &= \begin{cases} 1 \cdot (1 - \pi_{cg}) + e^{-\lambda_{cg}} \pi_{cg}, & \text{if } Y_{cg} = 0 \\ 0 \cdot (1 - \pi_{cg}) + \dfrac{\lambda_{cg}^{Y_{cg}} e^{-\lambda_{cg}}}{y_{cg}!} \pi_{cg}, & \text{if } Y_{cg} > 0 \end{cases} \\ &= \begin{cases} 1 + \pi_{cg}\left(e^{-\lambda_{cg}} - 1\right), & \text{if } Y_{cg} = 0 \\ \dfrac{\pi_{cg} \lambda_{cg}^{Y_{cg}} e^{-\lambda_{cg}}}{y_{cg}!}, & \text{if } Y_{cg} = 1 \end{cases}. \end{aligned} \tag{6}$$

Step.*e* Plug (1) and (2) into (6), the full likelihood of the spike-in RNA molecules can be expressed using the technical parameters $(\alpha_c, \beta_c, \kappa_c, \tau_c)$ and the amount of spike-in for g, μ_g,

$$\Pr[Y_{cg}] = \begin{cases} 1 + \text{expit}\left(\kappa_c + \tau_c \log \mu_g\right)\left(e^{-e^{\alpha_c + \beta_c \log \mu_g}} - 1\right), & \text{if } Y_{cg} = 0 \\ \dfrac{\text{expit}\left(\kappa_c + \tau_c \log \mu_g\right)\left[e^{\alpha_c + \beta_c \log \mu_g}\right]^{Y_{cg}} e^{-e^{\alpha_c + \beta_c \log \mu_g}}}{y_{cg}!}, & \text{if } Y_{cg} = 1 \end{cases}, \tag{7}$$

where

$$\text{expit}[x] = \frac{1}{1 + \exp[-x]}. \tag{8}$$

1.2 Empirical Bayes estimation of cell-specific technical parameters

Cell-specific technical parameters include

- α_c and β_c, characterizing capture and amplification efficiencies for any gene in cell c.
- κ_c and τ_c, characterizing the probability of any gene to be detected, *i.e.* not undetected due to technical dropout, in cell c.

From the generative model, we have arrived at the full marginal likelihood for Y_{cg} given the technical parameters $\mathbf{\Psi}_c = (\alpha_c, \beta_c, \kappa_c, \tau_c)$. Maximum likelihood estimates (MLEs) can be obtained by optimizing the complete likelihood over the support of $\mathbf{\Psi}_c$ for cell c. However, in our simulations, naïve MLEs suffer from numerical instability and lack of convergence for κ_c and τ_c (Figure S1), which prompts us to derive a better strategy for estimating κ_c and τ_c.

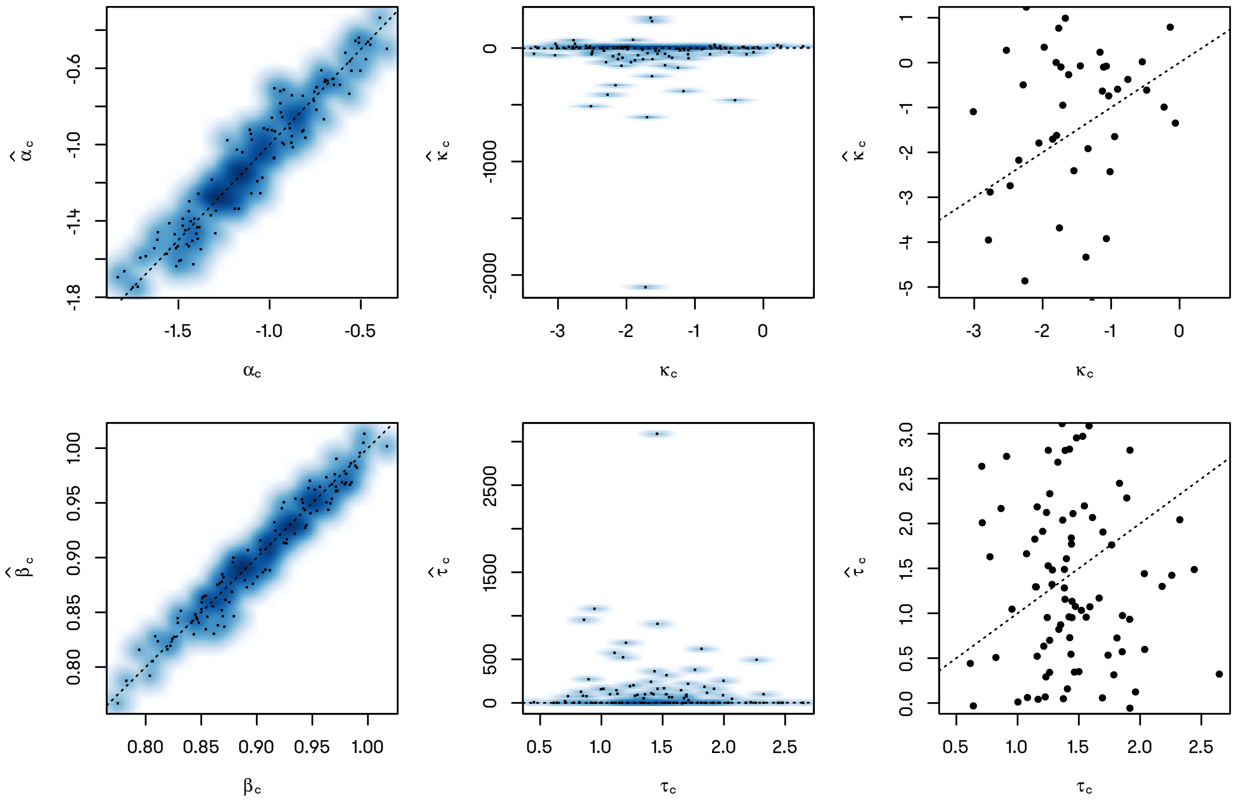

Figure S1: Comparing the maximum likelihood estimators of cell-specific technical parameters $\mathbf{\Psi}_c$ with their true values. Left panel: scatter plot comparing α_c (upper) and β_c (lower) estimated with maximum likelihood methods (y axis) to their true values (x axis). Middle panel: scatter plot comparing κ_c (upper) and τ_c (lower) estimated with maximum likelihood methods (y axis) to their true values (x axis). Right panel: scatter plot comparing κ_c (upper) and τ_c (lower) estimated with maximum likelihood methods (y axis) to their true values (x axis), zoomed in view. Identity line (dotted) is plotted for ease of comparison.

Upon further investigation, we have pinpointed the issues with likelihood estimators:

- due to the limitations of ERCC spike-ins, each cell contains little information w.r.t the drop-out probabilities due to paucity of spike-ins with low concentrations, thus necessitating borrowing information across cells if we wish to estimate the dropout-related parameters with better stability;
- we have observed that the κ_c and τ_c are negatively correlated, and similar relationships are observed for α_c and β_c as well (Figure S2). The estimating procedure can take advantage of this knowledge to model the correlation among the parameters.

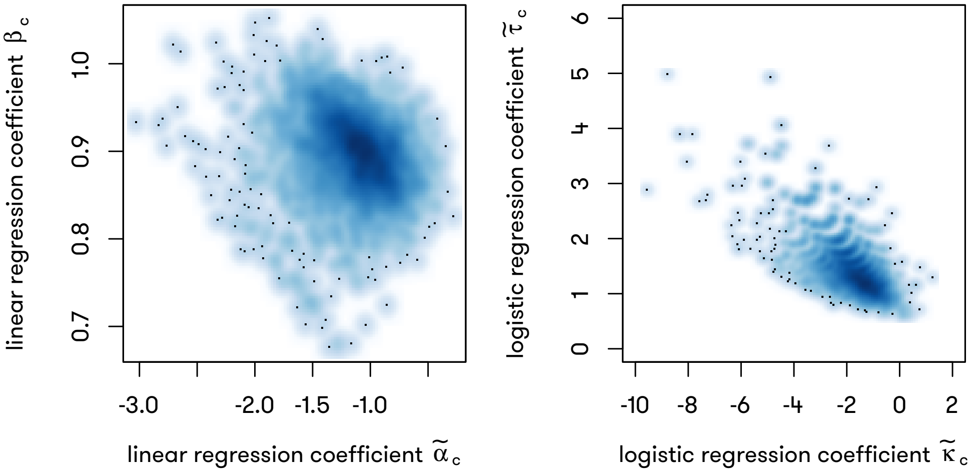

Figure S2: Scatter plot describing the correlation between α_c and β_c, and κ_c and τ_c. Left panel: $\tilde{\alpha}_c$ (y axis) compared to $\tilde{\beta}_c$ (x axis); both estimated from linear regressions. Right panel: $\tilde{\kappa}_c$ (y axis) compared to $\tilde{\tau}_c$ (x axis); both estimated from logistic regressions.

Taking the above observations into consideration, we propose an empirical Bayesian approach in which we assume the vector $\boldsymbol{\Psi}_c = (\alpha_c, \beta_c, \kappa_c, \tau_c)$ follows a multivariate normal distribution with mean ψ and covariance matrix $\Sigma_{\boldsymbol{\Psi}}$

$$\boldsymbol{\Psi}_c \sim \mathcal{N}(\psi, \Sigma_{\boldsymbol{\Psi}}). \tag{9}$$

Denote the observed read counts for the spike-in molecules as $\mathbf{Y}_c = \{Y_{cg}, g = 1, \cdots, G\}$, with G being the number of synthetic mRNA molecules added to the cell lysates. Assuming independence $Y_{c_1 g} \perp\!\!\!\perp Y_{c_2 g}$ for $c_1 \neq c_2$, the full likelihood for the observed Y_{cg} across cells can be written as,

$$\begin{aligned} \mathcal{L}[\psi, \Sigma_{\boldsymbol{\Psi}} | \mathbf{Y}] &= \prod_c \Pr[\mathbf{Y}_c | \psi, \Sigma_{\boldsymbol{\Psi}}] \\ &= \prod_c \int \Pr[\mathbf{Y}_c | \boldsymbol{\Psi}_c] \Pr[\boldsymbol{\Psi}_c | \psi, \Sigma_{\boldsymbol{\Psi}}] \, d\boldsymbol{\Psi}_c. \end{aligned} \tag{10}$$

Conditional on $\boldsymbol{\Psi}_c$, the probability density function of $\mathbf{Y}_c$ is just the likelihood in (7), and $\Pr[\boldsymbol{\Psi}_c | \psi, \Sigma_{\boldsymbol{\Psi}}]$ is the bivariate normal density function per our assumptions. To estimate the expected values of $\boldsymbol{\Psi}_c$ in the above models, we need to first compute the hyper-parameters $(\psi, \Sigma_{\boldsymbol{\Psi}})$ by maximizing the above likelihood. Due to the lack of closed form solutions, this calls for the numerical maximization of a numerically integrated function. The integration would be evaluated over 4 variables, and the maximization over 14 (4 for the mean and 10 for the covariance matrix) with a positive-definite restraint on $\Sigma_{\boldsymbol{\Psi}}$. This numerical problem has turned out to be unsolvable for the current computational infrastructure accessible by the majority of our users. We propose a computationally efficient approach to estimate the required parameters $\boldsymbol{\Psi}_c$ for all cells. We recognize that constraints on the covariance structure $\Sigma_{\boldsymbol{\Psi}}$ are necessary to reduce the dimensionality of our optimization. We assume $\Sigma_{\boldsymbol{\Psi}}$ is a diagonal block matrix by imposing independence between the vectors (α_c, β_c) and (κ_c, τ_c). Then we estimate these two vectors separately.

(α_c, β_c) can be estimated efficiently by fitting the linear regression with $\log[Y_{cg}]$ as the response variable and the amount of spiked-in ERCC molecules as the predictor variable, using only genes that are detected ($\{g, \text{s.t. } Y_{cg} > 0\}$),

$$\log E[Y_{cg}] = \alpha_c + \beta_c \log \mu_g. \tag{11}$$

We recognize that this estimator is biased as a result of the data missing not at random (MNAR). However, in our simulation studies, this estimator does not show any discernible bias when compared to the truth (Fig-S3), indicating the bias incurred by MNAR is under control.

On the other hand, the alternative estimators for (κ_c, τ_c) have proven to be a bit more elusive since the indicator of dropout is latent, *i.e.* we do not directly observe which zeros in our read counts are caused by technical dropouts *versus* Poisson sampling during sequencing. One approach is to assume all zeros are technical dropouts, and use logistic regression to estimate (κ_c, τ_c),

$$\text{logit}\left(\Pr[Y_{cg} > 0|\mu_g]\right) = \kappa_c + \tau_c \log \mu_g. \tag{12}$$

However, this has two drawbacks. First, this estimator is highly biased, since not all zeros are effects of technical dropout, and some of these zeros are due to the low expression of gene g in cell c. Second, since those genes with lower expression have a higher probability of dropping out, naïve logistic regressions could fail from complete or quasi-complete separation. Complete and quasi-complete separation happens when the outcome variable (in this case the event of being observed) separates a predictor (in this case $\log \mu_{cg}$) completely (complete) or very well to a certain extent (quasi-complete). In both cases, the coefficients associated with the affected covariates cannot be estimated. Our model requires that all of the cell-specific technical parameters be known for the downstream computations, failure to estimate (κ_c, τ_c) will result in cell c being removed from the sample pool, thus causing unnecessary loss of data. The root of this issue is identical to that of the simple MLEs (Figure S1), therefore similarly some form of shrinkage is the key to stably estimating these two dropout-related parameters.

We propose the following steps to compute the cell-specific dropout parameters (κ_c, τ_c). Let $\boldsymbol{\delta}_c = (\kappa_c, \tau_c)$.

- Step.*a* perform logistic regression of (12) and obtain $\hat{\delta}_c$ for cells that do not exhibit complete or quasi-complete separations.
- Step.*b* estimate prior of $\boldsymbol{\delta}_c$ by fitting a bivariate normal distribution using the estimated $\hat{\boldsymbol{\delta}}_c$ to compute the mean $E[\boldsymbol{\delta}_c]$ and covariance matrix $\Sigma_{\boldsymbol{\delta}_c}$.
- Step.*c* use the estimated mean and covariance matrix of $\boldsymbol{\delta}_c$ to compute the posterior mean of κ_c and τ_c. The complete probability density function for $\boldsymbol{\delta}_c$ and $\mathbf{Y}_c$ is

$$\begin{aligned}\Pr[\boldsymbol{\delta}_c, \mathbf{Y}_c] &= \Pr[\mathbf{Y}_c|\boldsymbol{\delta}_c]\Pr[\boldsymbol{\delta}_c] \\ &= \Pr[\boldsymbol{\delta}_c]\prod_g \Pr[Y_{cg}|\boldsymbol{\delta}_c] \\ &= f_N(\boldsymbol{\delta}_c|E[\boldsymbol{\delta}_c], \Sigma_{\boldsymbol{\delta}_c}) \cdot \prod_g \Pr[Y_{cg}|\boldsymbol{\delta}_c].\end{aligned} \tag{13}$$

The posterior distribution of δ_c is

$$\Pr[\delta_c|\mathbf{Y}_c] = \frac{\Pr[\boldsymbol{\delta}_c, \mathbf{Y}_c]}{\Pr[\mathbf{Y}_c]} = \frac{\Pr[\boldsymbol{\delta}_c, \mathbf{Y}_c]}{\int \Pr[\boldsymbol{\delta}_c, \mathbf{Y}_c]\, d\boldsymbol{\delta}_c}, \tag{14}$$

with f_N being the PDF of a bivariate normal density and $\Pr[Y_{cg}|\delta_c]$ is equal to (7) in form. The posterior mean of $\boldsymbol{\delta}_c$ can then be computed by integrating the PDF over the support of bivariate random variable $\boldsymbol{\delta}_c$, *i.e.* $\mathbb{R}^2$.

$$\begin{aligned} E[\kappa_c|\mathbf{Y}_c] &= \int \kappa_c \Pr[\boldsymbol{\delta}_c|\mathbf{Y}_c]\, d\boldsymbol{\delta}_c \\ &= \int \frac{\kappa_c \Pr[\boldsymbol{\delta}_c, \mathbf{Y}_c]}{\int \Pr[\boldsymbol{\delta}_c, \mathbf{Y}_c]\, d\boldsymbol{\delta}_c} d\boldsymbol{\delta}_c \\ &= \frac{\int \kappa_c \Pr[\boldsymbol{\delta}_c, \mathbf{Y}_c]\, d\boldsymbol{\delta}_c}{\int \Pr[\boldsymbol{\delta}_c, \mathbf{Y}_c]\, d\boldsymbol{\delta}_c} \end{aligned} \tag{15}$$

$$E[\tau_c|\mathbf{Y}_c] = \frac{\int \tau_c \Pr[\boldsymbol{\delta}_c, \mathbf{Y}_c]\, d\boldsymbol{\delta}_c}{\int \Pr[\boldsymbol{\delta}_c, \mathbf{Y}_c]\, d\boldsymbol{\delta}_c} \tag{16}$$

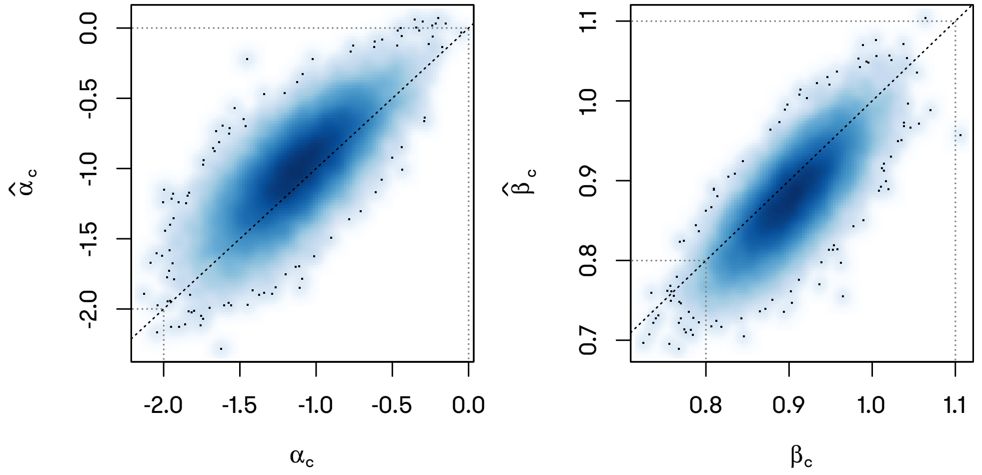

Figure S3: Comparing the estimated $\hat{\alpha}_c$, $\hat{\beta}_c$ to the true values of α_c and β_c. Left panel: $\hat{\alpha}$ estimated from linear regressions (y axis) compared to their true values (x axis). Right panel: $\hat{\beta}$ estimated from linear regressions (y axis) compared to their true values (x axis). Both panels: dotted lines represent the unit lines with intercept equal to 0, and slope equal to 1.

We have performed a series of simulation studies to assess the performance of the aforementioned estimators of α_c, β_c, κ_c and τ_c. Using the largest level 2 class in the Zeisel data[1], we have estimated the cell-specific parameters (α_c, β_c) and (κ_c, τ_c) using the method described above. Denote $\zeta_c = (\alpha_c, \beta_c)$. Two bivariate normal distributions are fitted to the estimated parameters $\hat{\zeta}_c$ and $\hat{\boldsymbol{\delta}}_c$ to get the mean and covariance matrices of these two vectors, $E[\zeta_c]$, Σ_ζ, $E[\boldsymbol{\delta}_c]$, and Σ_δ. New technical parameters are then sampled from the bivariate normal distributions $\mathcal{N}(E[\zeta_c], \Sigma_\zeta)$ and $\mathcal{N}(E[\boldsymbol{\delta}_c], \Sigma_\delta)$. From these new technical parameters, counts of 57

ERCC spike-ins present in the Zeisel data[1] in 200 cells are generated according to the hierarchical model described in the main text. The simulation is repeated 100 times to get a more comprehensive picture of the performance of these estimators.

Despite being biased, the linear estimator for ζ_c has performed fairly well showing high concordance with the truth (Figure S3). As expected, estimated β_c is slightly lower than true β_c, and estimated α_c is slightly lower than true α_c. However, in general the true value can be efficiently recovered even in the presence of this minor yet discernible bias.

The empirical Bayesian estimator for δ_c has also displayed decent concordance with the truth (Figure S4). More importantly, when compared to the naïve logistic regressions, our empirical Bayesian estimators show dramatic improvement in terms of accuracy. Estimates from naïve logistic regressions show a much larger spread, and this is after we have filtered out a significant portion of the cells showing complete or quasi-complete separation, in which case the estimates cannot be obtained at all. These samples would need to be discarded in downstream analyses if no shrinkage is implemented.

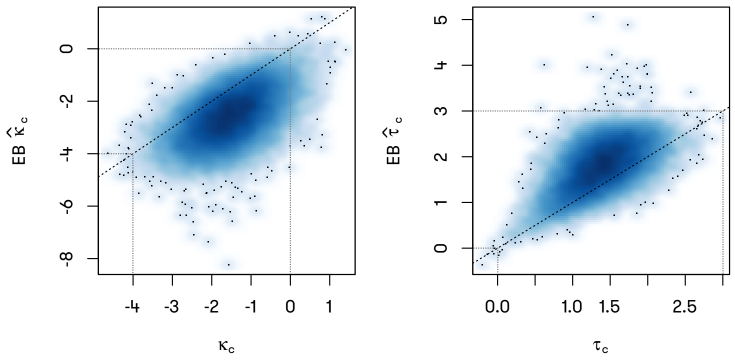

(a) $\hat{\kappa}_c$ (left panel) and $\hat{\kappa}_c$ (right panel) estimated using the empirical Bayes approach compared to their true values.

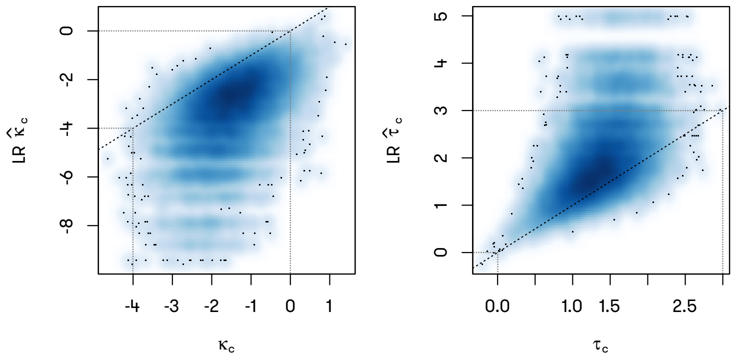

(b) $\hat{\kappa}_c$ (left panel) and $\hat{\kappa}_c$ (right panel) estimated using simple logistic regressions compared to their true values.

Figure S4: Comparing the estimated $\hat{\kappa}_c$, $\hat{\tau}_c$ to the true values of κ_c and τ_c. Dotted line represents the unit line with intercept being 0, and slope equal to 1.

1.3 Modelling biological genes

The true expression of biological genes vary amongst cells.[2] Thus, its value is sampled from a gene-specific distribution F_g in our framework. Assuming F_g is in the form of log-normal distribution, the count of reads or transcripts for a biological gene g in cell c can be modelled with the following steps:

Step.*a* Given the cell-specific technical parameters, we assume the actual expression of gene g in cell c follows[3],

$$\mu_{cg} \sim \text{LogNormal}\left(\theta_g, \sigma_g^2\right), \tag{17}$$

where θ_g and σ_g are the gene-specific parameters characterizing the mean and standard deviation of the log-normal distribution.

Step.*b* Let Z_{cg} be the indicator that dropout does not occur. The probability of $Z_{cg} = 1$ (π_{cg}) depends on the gene's true absolute expression in the cell, μ_{cg}. A logistic model can be used to describe this relationship.

$$\begin{aligned} \pi_{cg} =& \operatorname{expit}\left[\kappa_c + \tau_c \log \mu_{cg}\right] \\ Z_{cg}|\mu_{cg} \sim& \text{Bernoulli}\left(\pi_{cg}\right) \end{aligned} \tag{18}$$

Step.*c* Given the cell-specific technical parameters $(\alpha_c, \beta_c, \kappa_c, \tau_c)$, let λ_{cg} be the expected value for the read count of spike-in g in cell c.

$$\log \lambda_{cg} = \alpha_c + \beta_c \log \mu_{cg} \tag{19}$$

Step.*d* Similar to the case of spike-in molecules, given the status of Z_{cg}, the observed count for gene g in cell c, Y_{cg}, can be modelled as,

$$Y_{cg}|Z_{cg}, \mu_{cg} = \begin{cases} \text{Poisson}(\lambda_{cg}), & \text{if } Z_{cg} = 1 \\ 0, & \text{if } Z_{cg} = 0 \end{cases}. \tag{20}$$

And the conditional probability density function is,

$$\begin{aligned} \Pr\left[Y_{cg}|Z_{cg} = 0, \mu_{cg}\right] =& \begin{cases} 1, & \text{if } Y_{cg} = 0 \\ 0, & \text{if } Y_{cg} > 0 \end{cases} \\ \Pr\left[Y_{cg}|Z_{cg} = 1, \mu_{cg}\right] =& \frac{\lambda_{cg}^{Y_{cg}} e^{-\lambda_{cg}}}{y_{cg}!} \\ =& \frac{\left[e^{\alpha_c + \beta_c \log \mu_{cg}}\right]^{Y_{cg}} e^{-e^{\alpha_c + \beta_c \log \mu_{cg}}}}{y_{cg}!}. \end{aligned}$$

Step.*e* The joint probability of Y_{cg}, Z_{cg} and μ_{cg} can be subsequently expressed as,

$$\begin{aligned} \Pr\left[Y_{cg}, Z_{cg}, \mu_{cg}\right] =& \Pr\left[Y_{cg}|Z_{cg}, \mu_{cg}\right] \Pr\left[Z_{cg}, \mu_{cg}\right] \\ =& \Pr\left[Y_{cg}|Z_{cg}, \mu_{cg}\right] \Pr\left[Z_{cg}|\mu_{cg}\right] \Pr\left[\mu_{cg}\right]. \end{aligned}$$

The marginal likelihood of Y_{cg}, μ_{cg} can be computed by summing over the support of Z_{cg},

$$\begin{aligned}
&\Pr[Y_{cg}, \mu_{cg}] \\
=&\Pr[Y_{cg}, \mu_{cg}, Z_{cg} = 0] + \Pr[Y_{cg}, \mu_{cg}, Z_{cg} = 1] \\
=&\Pr[Y_{cg}|Z_{cg} = 0, \mu_{cg}] \Pr[Z_{cg} = 0|\mu_{cg}] \Pr[\mu_{cg}] \\
&+ \Pr[Y_{cg}|Z_{cg} = 1, \mu_{cg}] \Pr[Z_{cg} = 1|\mu_{cg}] \Pr[\mu_{cg}] \\
=&\begin{cases} (1-\pi_{cg}) f_{LN}\left(\mu_{cg}|\theta_g, \sigma_g^2\right) + e^{-e^{\alpha_c+\beta_c \log \mu_{cg}}} \pi_{cg} f_{LN}\left(\mu_{cg}|\theta_g, \sigma_g^2\right), & \text{if } Y_{cg} = 0 \\ \dfrac{\left[e^{\alpha_c+\beta_c \log \mu_{cg}}\right]^{Y_{cg}} e^{-e^{\alpha_c+\beta_c \log \mu_{cg}}}}{y_{cg}!} \pi_{cg} f_{LN}\left(\mu_{cg}|\theta_g, \sigma_g^2\right), & \text{if } Y_{cg} > 0 \end{cases},
\end{aligned} \tag{21}$$

where

$$f_{LN}\left(\mu_{cg}|\theta_g, \sigma_g^2\right) = \frac{1}{\mu_{cg}\sigma_g\sqrt{2\pi}} e^{-\frac{(\ln \mu_{cg}-\theta_g)^2}{2\sigma_g^2}}. \tag{22}$$

Therefore, the marginal likelihood for Y_{cg} can be computed by integrating out μ_{cg},

$$\Pr[Y_{cg}] = \int_{\mu_{cg}} \Pr[Y_{cg}, \mu_{cg}]\, d\mu_{cg}.$$

Assuming independence between cells, then the marginal distribution of $\mathbf{Y}_g = (Y_{1g}, \cdots, Y_{cg}, \cdots, Y_{Ng})$ can be expressed as,

$$\Pr[\mathbf{Y}_g] = \prod_{c=1}^{N} \int_{\mu_{cg}} \Pr[Y_{cg}, \mu_{cg}]\, d\mu_{cg}. \tag{23}$$

The parameters θ_g and σ_g^2 can therefore be estimated by maximizing the above marginal likelihood.

The choice of the distributional form of μ_{cg} can be extended to a zero-inflated log-normal distribution, in order to specifically model transcriptional bursting. This extension however in our simulation is not necessary if one simply wishes to estimate the mean expression of the genes of interest, as is displayed in Figure S5. In this study, the estimated mean expression from TASC (labeled TASC) and the estimated posterior mean from TASC with a zero-inflated log-normal distribution (TASC-B) are highly correlated for the majority of the genes. Difference only occurs when the expression of the genes is very low, where the extended Log-normal distribution holds an advantage as it more thoroughly models the inflated zeros.
This comparison is performed with level 2 class “CA1Pyr2” from Zeisel et al. data. The expression of 5000 genes randomly picked from this dataset is computed with the two methods above.. Estimates from the original TASC, with μ_{cg} following a log-normal distribution are computed by numerically maximizing the numerically integrated marginal likelihood using the TASC implementation reported in this manuscript. Estimates from the extended TASC (TASC-B), with μ_{cg} following a zero-inflated log-normal distribution are computed by calculating the mean of the parameters sampled from the posterior distribution with MCMC using Stan.

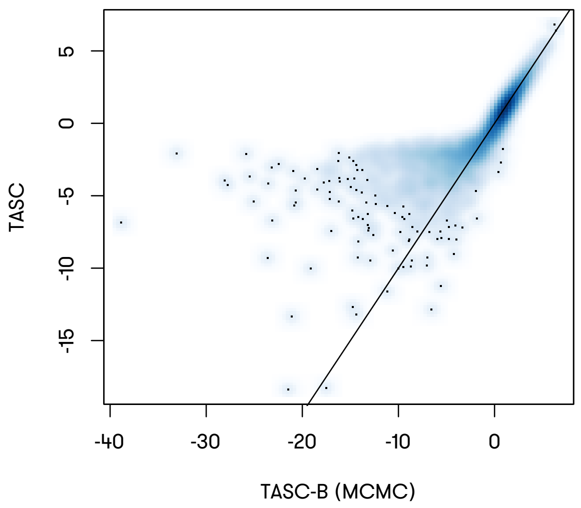

Figure S5: Scatter plot comparing estimates of mean expression using the TASC models with $\mathcal{F}$ following log-normal distribution (TASC) and zero-inflated log-normal distribution (TASC-B). The former is fitted with C++ implemented TASC, and the latter is fitted with MCMC using PyStan and MPI4Py.

1.4 Differential expression analysis

Based on the hierarchical model, testing for differentially expressed (DE) genes is straightforward. In our model, for cells within the same group, the true expression level of gene g, μ_{cg} follows a log-normal distribution with mean θ_g and variance σ_g^2. Testing for differential expression involves comparing the means from different groups on a gene-by-gene basis. We propose a likelihood ratio test for this purpose. Let θ_g be expressed as a linear combination of the covariates for which one wishes to test or adjust, $\theta_g = X\Gamma_g$, $\Gamma_g = (\gamma_1, \cdots, \gamma_i, \cdots, \gamma_p)$ with γ_i denoting the coefficient for predictor x_i in the design matrix $X = (x_1, \cdots, x_i, \cdots, x_p)$. Testing for each covariate involves fitting a full model $\theta_g = X\Gamma_g$ and a reduced model with target covariate x_i removed from the design matrix X. Denote the reduced design matrix and coefficient vector to be $\tilde{X}$ and $\tilde{\Gamma}_g$ respectively. Denote the biological variance of the full and reduced model as σ_g^2 and $\tilde{\sigma}_g^2$. Formally the question of whether x_i is significantly associated with the gene expression can be formulated as the following hypothesis test,

$$H_0 : \gamma_i = 0$$
$$H_1 : \gamma_i \neq 0.$$

The likelihood ratio test statistic for this above test, T_i can be constructed as,

$$T_{ig} = 2\left[\log\left(\hat{\mathcal{L}}_1\right) - \log\left(\hat{\mathcal{L}}_0\right)\right],$$

where $\hat{\mathcal{L}}_1$ and $\hat{\mathcal{L}}_0$ are the likelihoods maximized under H_1 and H_0, respectively. Asymptotically, $T_{ig}i$ follows a χ^2-distribution with 1 degree of freedom under the null hypothesis ($\gamma_i = 0$). Raw p-values can subsequently be adjusted for multiple comparisons with false discovery rate controlling procedures such as the Benjamini-Hochberg procedure or the Holm-Bonferroni procedure.

1.5 Expectation-Maximization algorithm

When the number of covariates is small, the parameters can be estimated using the Simplex algorithm, which does not involve the calculation of derivatives. However, the Simplex algorithm is not suitable when the number of covariates is large. To circumvent this problem, we have also developed an expectation-maximization (EM) algorithm to estimate the biological mean (θ_g) and variance σ_g^2. Briefly, the log likelihood for gene g can be written as,

$$\begin{aligned}
\ell\left[\mathbf{Y}_g, \boldsymbol{\mu}_g|\theta_g, \sigma_g^2\right] &= \sum_c \ell\left[Y_{cg}, \mu_{cg}|\theta_g, \sigma_g^2\right] \\
&= \sum_c \left\{\ell\left[Y_{cg}|\mu_{cg}, \theta_g, \sigma_g^2\right] + \ell\left[\mu_{cg}|\theta_g, \sigma_g^2\right]\right\} \\
&= \sum_c \left\{\ell\left[Y_{cg}|\mu_{cg}\right] + \ell\left[\mu_{cg}|\theta_g, \sigma_g^2\right]\right\} \\
&= \sum_c \ell\left[Y_{cg}|\mu_{cg}\right] + \sum_c \ell\left[\mu_{cg}|\theta_g, \sigma_g^2\right].
\end{aligned}$$

E-step:

$$\begin{aligned}
&\mathsf{E}\left[\ell\left[\mathbf{Y}_g, \boldsymbol{\mu}_g\middle|\,\theta_g, \sigma_g^2\right]|\hat{\theta}_g^{(t)}, \hat{\sigma}_g^{(t)}, \mathbf{Y}_g\right] \\
=&\sum_c \mathsf{E}\left[\ell\left[Y_{cg}|\mu_{cg}\right]|\,\hat{\theta}_g^{(t)}, \hat{\sigma}_g^{(t)}, \mathbf{Y}_g\right] + \sum_c \mathsf{E}\left[\ell\left[\mu_{cg}|\theta_g, \sigma_g^2\right]|\,\hat{\theta}_g^{(t)}, \hat{\sigma}_g^{(t)}, \mathbf{Y}_g\right] \\
=&\mathsf{C}(\hat{\theta}_g^{(t)}, \hat{\sigma}_g^{(t)}) + \sum_c \mathsf{E}\left[\ell\left[\mu_{cg}|\theta_g, \sigma_g^2\right]|\,\hat{\theta}_g^{(t)}, \hat{\sigma}_g^{(t)}, \mathbf{Y}_g\right]
\end{aligned}$$

The first term is ignorable since it is a constant function of $\hat{\theta}_g^{(t)}, \hat{\sigma}_g^{(t)}$, over which the maximization is to be performed. So in order to evaluate this expectation in the E-step, we only need to compute $\mathsf{E}\left[\ell\left[\mu_{cg}|\theta_g, \sigma_g^2\right]|\,\hat{\theta}_g^{(t)}, \hat{\sigma}_g^{(t)}, \mathbf{Y}_g\right]$. Due to the assumptions we have made for the functional form of F_g, it follows a log normal distribution.

$$\begin{aligned}
&\mathsf{E}\left[\ell\left[\mu_{cg}|\theta_g, \sigma_g^2\right]|\hat{\theta}_g^{(t)}, \hat{\sigma}_g^{(t)}, \mathbf{Y}_g\right] \\
=&\mathsf{E}\left\{\left[-\frac{[\log(\mu_{cg}) - \theta_g]^2}{2\sigma_g^2} - \frac{1}{2}\log\left(2\pi\sigma_g^2\right)\right]\middle|\,\hat{\theta}_g^{(t)}, \hat{\sigma}_g^{(t)}, \mathbf{Y}_g\right\} \\
=&\mathsf{E}\left\{\left[-\frac{\log(\mu_{cg})^2 - 2\theta_g\log(\mu_{cg}) + \theta_g^2}{2\sigma_g^2} - \frac{1}{2}\log\left(2\pi\sigma_g^2\right)\right]\middle|\,\hat{\theta}_g^{(t)}, \hat{\sigma}_g^{(t)}, \mathbf{Y}_g\right\} \\
=&-\frac{\mathsf{E}\left[\log(\mu_{cg})^2|\hat{\theta}_g^{(t)}, \hat{\sigma}_g^{(t)}, \mathbf{Y}_g\right] - 2\hat{\theta}_g^{(t)}\mathsf{E}\left[\log(\mu_{cg})|\hat{\theta}_g^{(t)}, \hat{\sigma}_g^{(t)}, \mathbf{Y}_g\right] + \left(\hat{\theta}_t^{(t)}\right)^2}{2\left(\hat{\sigma}_g^{(t)}\right)^2} - \frac{1}{2}\log\left(2\pi\left(\hat{\sigma}_g^{(t)}\right)^2\right)
\end{aligned}$$

Two expectations need to be evaluated in order to compute the above value. Briefly,

$$E\left[\log(\mu_{cg})^2 \,|\hat{\theta}_g^{(t)}, \hat{\sigma}_g^{(t)}, \mathbf{Y}_g\right] = \frac{\int_0^\infty \log[\mu_{cg}]^2 \Pr[Y_{cg}|\mu_{cg}] \Pr\left[\mu_{cg}|\hat{\theta}_g^{(t)}, \hat{\sigma}_g^{(t)}\right] d\mu_{cg}}{\int_0^\infty \Pr[Y_{cg}|\mu_{cg}] \Pr\left[\mu_{cg}|\hat{\theta}_g^{(t)}, \hat{\sigma}_g^{(t)}\right] d\mu_{cg}}$$

$$E\left[\log(\mu_{cg}) \,|\hat{\theta}_g^{(t)}, \hat{\sigma}_g^{(t)}, \mathbf{Y}_g\right] = \frac{\int_0^\infty \log[\mu_{cg}] \Pr[Y_{cg}|\mu_{cg}] \Pr\left[\mu_{cg}|\hat{\theta}_g^{(t)}, \hat{\sigma}_g^{(t)}\right] d\mu_{cg}}{\int_0^\infty \Pr[Y_{cg}|\mu_{cg}] \Pr\left[\mu_{cg}|\hat{\theta}_g^{(t)}, \hat{\sigma}_g^{(t)}\right] d\mu_{cg}}$$

M-step:
The M-step involves maximizing the above expected log-likelihood w.r.t the parameters $\hat{\theta}_g^{(t)}$ and $\hat{\sigma}_g^{(t)}$, in the case of simple quantification,

$$\hat{\theta}_g^{(t+1)} = \frac{1}{N}\sum_{c=1}^{N} E\left[\log(\mu_{cg}) \,|\hat{\theta}_g^{(t)}, \hat{\sigma}_g^{(t)}, \mathbf{Y}_g\right]^{(t)}$$

$$\hat{\sigma}_g = \sqrt{\frac{1}{N}\sum_{c=1}^{N} E\left[\log(\mu_{cg})^2 \,|\hat{\theta}_g^{(t)}, \hat{\sigma}_g^{(t)}, \mathbf{Y}_g\right] - 2\hat{\theta}_g^{(t+1)} E\left[\log(\mu_{cg}) \,|\hat{\theta}_g^{(t)}, \hat{\sigma}_g^{(t)}, \mathbf{Y}_g\right] + \left(\hat{\theta}_t^{(t+1)}\right)^2}.$$

In the case of $\theta_g = X\Gamma_g$, the above E-step is the same, after substituting $\hat{\theta}_g^{(t)} = X\hat{\Gamma}_g^{(t)}$. The M-step for $\hat{\Gamma}_g^{(t+1)}$ is replaced by a linear regression with $E\left[\log(\mu_{cg}) \,|\hat{\theta}_g^{(t)}, \hat{\sigma}_g^{(t)}, \mathbf{Y}_g\right]$ as the response variable, and X as the predictor. The M-step for $\hat{\sigma}_g^{(t+1)}$ is unchanged, after substituting $\hat{\theta}_g^{(t+1)} = X\hat{\Gamma}_g^{(t+1)}$.

1.6 Estimation of cell size factor

Single-cell RNA-seq requires normalization on cell size because larger cells tend to have more RNA molecules. To estimate the cell size S_c ($c = 1, \cdots, N$, N being the number of cells), we take advantage of the spike-ins as well. Denote the read count for biological gene b in cell c as $\xi_{cb}, b = 1, \cdots, B$, where B is the total number of biological genes after filtering. Also denote the counts of the spike-in molecule e as $\xi_{ce}, e = 1, \cdots, E$, where E is the total number of spike-in molecules. The cell size factor can be computed as,

$$S_c = \frac{\sum_{b=1}^{B} \xi_{cb}}{\sum_{e=1}^{E} \xi_{ce}}.$$

In our software implementation, this cell size factor is computed and automatically used as a covariate to adjust for any possible confounding incurred due to different cell sizes unless the users explicitly disable it. In order to compare the detected DE genes with and without adjustment for the cell size factors, we have looked at the genes called significantly differentially expressed when comparing the two level-2 classes CA1Pyr1 and CA1Pyr2. 1604 genes are uniquely detected with adjustment for the cell size factors, while 663 genes are uniquely detected without.

3346 genes are differentially expressed regardless of the adjustment. Considering the fact that this dataset contains cells of relatively homogeneous sizes (Figure S27), it is highly possible that the difference will be more pronounced in samples of more heterogeneous sizes.

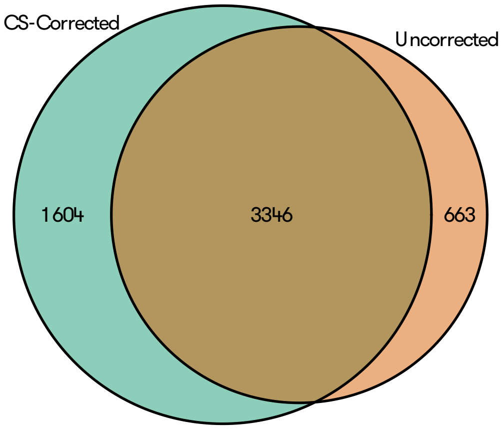

Figure S6: Venn diagram showing the overlapping of genes detected to be differentially expressed between comparisons with and without cell size adjustment.

2 Evaluation of Performance and Comparison with Other Methods

2.1 Type I error rates

In order to evaluate the specificity of TASC in the most realistic setting, we have performed differential expression analysis using the level-2 class "CA1Pyr2" from the Zeisel data[1]. "CA1Pyr2" contains 447 cells in total, and is shown to be homogeneous in their transcriptomic profiles. **We have randomly divided this class into two groups of roughly equal size**. Therefore, no gene should be differentially expressed when one group is compared with the other. We have performed differential expression analyses with the following packages, TASC, SCDE[4], MAST[5] and DESeq2[6]. Raw p-values are computed and histograms (main text Figure 4A) and Q-Q plots on the log scale (main text Figure 4B) are plotted for each method above.

2.2 Impact of batch effects

In scRNA-seq experiments, four technical parameters dictate the relationship between the true expression of a gene and the observed counts in a specific cell. In our framework, these four parameters are modelled in groups of two. The first two parameters are α_c and β_c, which represent the efficiency of capture and amplification, relating the log mean of the Poisson distribution to the true log expression of the gene in cell c. The last two parameters are κ_c and τ_c, which are influenced by the propensity of a gene being observed in the final sequencing, *i.e.* not a dropout. Both of these parameters vary across cells, and directly affect our estimates for the true expression of the gene g in cell c. Therefore, it is of great interest to see whether adjustment for these cell-specific technical parameters or failure to do so has an effect on the specificity for

calling significant differentially expressed genes from scRNA-seq data. Data with batch effects are generated through the following steps:

- Cell-specific parameters, $\boldsymbol{\Psi}_c$, as well as gene-specific parameters, the biological mean (θ_g) and variance (σ_g^2) are estimated from the "CA1Pyr2" class in the Zeisel data[1] using our model.
- Two bivariate normal distributions for $\delta_c = (\kappa_c, \tau_c)$ and $\zeta_c = (\alpha_c, \beta_c)$ are fitted with the estimated parameters.
- The sample is randomly divided into two groups of roughly equal sizes. A difference is then added to the mean of δ_c for cells from one of the groups, resulting in two sets of cells whose δ_c can be characterized as:

$$\begin{aligned} \mathrm{E}[\kappa_c]_2 =& \mathrm{E}[\kappa_c]_1 + \Delta\mathrm{E}[\kappa_c] \\ \mathrm{E}[\tau_c]_2 =& \mathrm{E}[\tau_c]_1 + \Delta\mathrm{E}[\tau_c] \end{aligned}$$

  The magnitudes of $\Delta\mathrm{E}[\kappa_c]$ and $\Delta\mathrm{E}[\tau_c]$ determine the degree of batch effects. We have generated combinations of $\Delta\mathrm{E}[\kappa_c]$ and $\Delta\mathrm{E}[\tau_c]$, with both values ranging from -0.4 to 0.8.
- The generative model is used to simulate the counts with $\boldsymbol{\delta}_c$ sampled from the corresponding bivariate normal distribution. For each combination of $\Delta\mathrm{E}[\kappa_c]$ and $\Delta\mathrm{E}[\tau_c]$, approximately 4000 genes are generated, and p-values are calculated by running differential expression analyses with each tested method.
- The p-values are subsequently used to compute the false positive rates (FPRs), *i.e.* the proportion of DE genes called ($p < 0.05$) among all genes tested (since all of them are not differentially expressed). The FPR is then compared with the desired significance level (0.05) and a heat map is generated by plotting $\log_{10}(\mathrm{FPR}/0.05)$ with varying colours on a grid representing the combinations of $\Delta\mathrm{E}[\kappa_c]$ and $\Delta\mathrm{E}[\tau_c]$.
- Similar simulations are performed for α_c and β_c, with the only change being the range of $\Delta\mathrm{E}[\alpha_c]$ ($[-1, 1]$) and $\Delta\mathrm{E}[\beta_c]$ ($[-0.1, 0.1]$).

2.3 Power

In addition to controlling type I error, an ideal statistical method should also be sensitive, *i.e.* exhibiting extraordinary power when compared to existing methods. In order to appraise the power of our framework in the most thorough and realistic fashion, we have devised the following scheme of simulations.

- The simulation scenario is the classic two-group comparison. Let the true expression of gene g from group 1 follow a log-normal distribution $\mu_{cg} \sim \mathrm{LogNormal}\left(\theta_{g1}, \sigma_g^2\right)$, and the same gene from group 2 follow a log-normal distribution with a different mean $\mu_{cg} \sim \mathrm{LogNormal}\left(\theta_{g2}, \sigma_g^2\right)$. For simplicity, in this simulation we assume g display similar biological variance across groups. This assumption is purely for simplicity, and our model can easily handle situations where this is not true. In our current iteration of implementation, the biological variance of the two groups is assumed to be identical.

- From cells in the level 2 class "CA1Pyr2" in Zeisel data set[1], we estimate the technical parameters $\mathbf{\Psi}_c$ for each cell c, the mean (θ_g) and standard deviation (σ_g) of log gene expression for reach gene g using TASC. Genes with extremely low total read counts are removed, leaving a total of 5018 genes in the final pool.

- 1000 genes are randomly picked to be differentially expressed. The effect size, *i.e.* fold change between the two groups, $\eta_g = \exp(|\theta_{g1} - \theta_{g2}|)$ ranges from 1.05 to 2.5, and is assigned so that the majority of DE genes only exhibit mild difference in expression (Figure S8). This distribution of η_g dovetails with the overall experience from two-group comparison experiments.

- Counts of the 1000 DE genes are sampled from our generative model using the technical parameters $\mathbf{\Psi}_c$ and σ_g^2 estimated in previous steps. More specifically, θ_{g1} is directly from the mean estimated in previous steps, and $\theta_{g2} = \theta_{g1} \pm \log \eta_g$, where η_g is the fold change for gene g. The sign of $\log \eta_g$ is randomly assigned.

- Counts of the 4018 non-DE genes are equal to the Zeisel data[1]. Since our group membership is randomly assigned, none of these genes should be differentially expressed.

- The above steps are repeated 100 times and each dataset consists of 5018 genes (1000 DE genes and 4018 non-DE genes). The 447 cells are then down-sampled into various sample sizes for 5 different simulations, 20 vs 20 (20 cells in group 1 and 20 cells in group 2, same hereinafter), 50 vs 50, 100 vs 100, 150 vs 150 and 200 vs 200.

- In each simulation, TASC, MAST[5] and DESeq2[6] are used to call DE genes. For each DE gene, the power can be estimated by dividing the number of datasets in which it is called significant (p is less than or equal to the pre-set significant level) by the total number of simulations (100).

The scheme of simulation is illustrated in Figure S7.

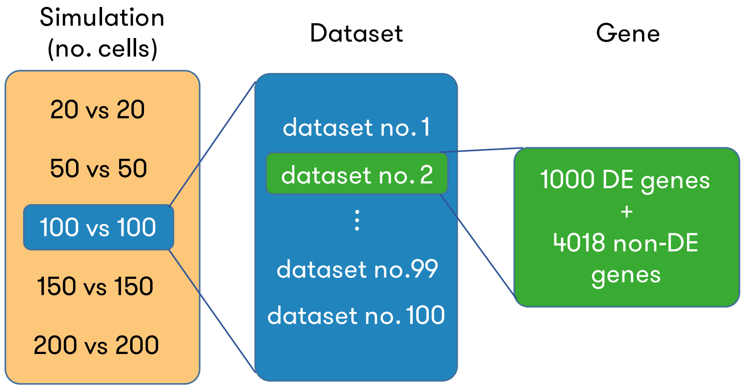

Figure S7: The scheme of simulation for power comparisons. Simulations differ by their sample sizes, *i.e.* the number of cells in each group. This is achieved by downsampling each group to the desired number of cells from the complete data (447 cells in total). One simulation contains 100 datasets, generated by repeating the sampling process from the same parameters. Each dataset contains the counts of 5018 genes in specified number of cells. 1000 genes are differentially expressed while the rest are not.

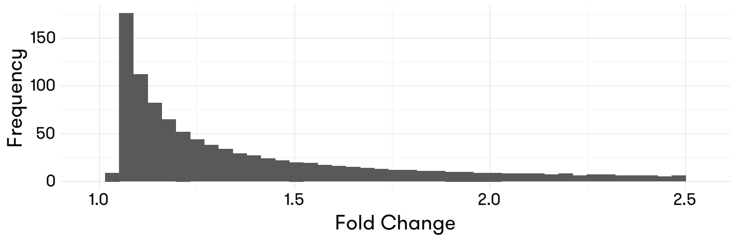

Figure S8: Distribution of η_g in the simulation study.

We have made sure that our simulated datasets are visually indistinguishable when counts from a random pair of cells are compared. In Figure S9, 9 pairs of cells from the 447 cells are randomly selected and plotted. In a specific pair, each dot represents a gene with its count in one cell plotted on the x axis and that in the other cell on the y axis. These plots closely resemble similar plots reported before generated from various scRNA-seq experiments, which suggests that our simulation scheme can largely recapitulate the between cell variability in scRNA-seq data.

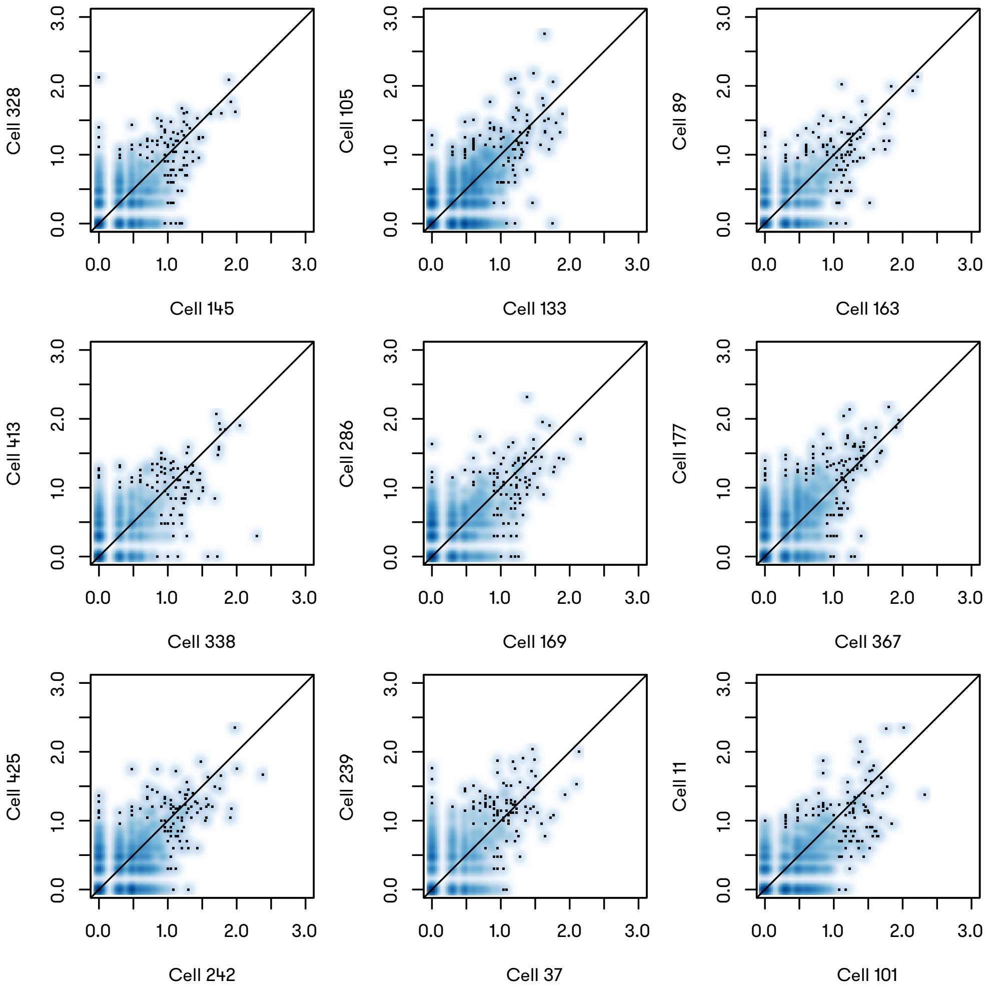

Figure S9: Scatter plots for 9 randomly picked pairs of cells in simulated data. For each panel, two cells are randomly chosen from the a total of 447. With two cells indexed as i and j, $\log(Y_{ig} + 1)$ is plotted against $\log(Y_{jg} + 1)$.

2.3.1 Power and effect size

Since we have simulated 1000 DE genes with varying effect size, it is straightforward to investigate how the η_g influences the power of our method. In Figure S10, estimated power ($\omega_g = n_{Sg}/n_{Tg}$, where n_{Sg} is the number of datasets in which the p-value of TASC is less than or equal to the specified significance level, and $n_{Tg} = 100$ is the total number of datasets in each simulation) is plotted against η_g. Due to the differences in other parameters such as θ_{g1}, θ_{g2} and σ_g, genes with similar η_g can be detected with dramatically different power. This leads to a spread in our power-effect size curve. For example, when we pick the significance level to be 10^{-4}, genes that display approximately 2-fold change between the two groups can be detected from less than 40% of the time to over 80% depending on specific properties of the gene. This closely resembles the actual analyses and speaks to the importance of simulating data based on real data. Figure S10 is plotted from the simulation with 100 cells in each group.

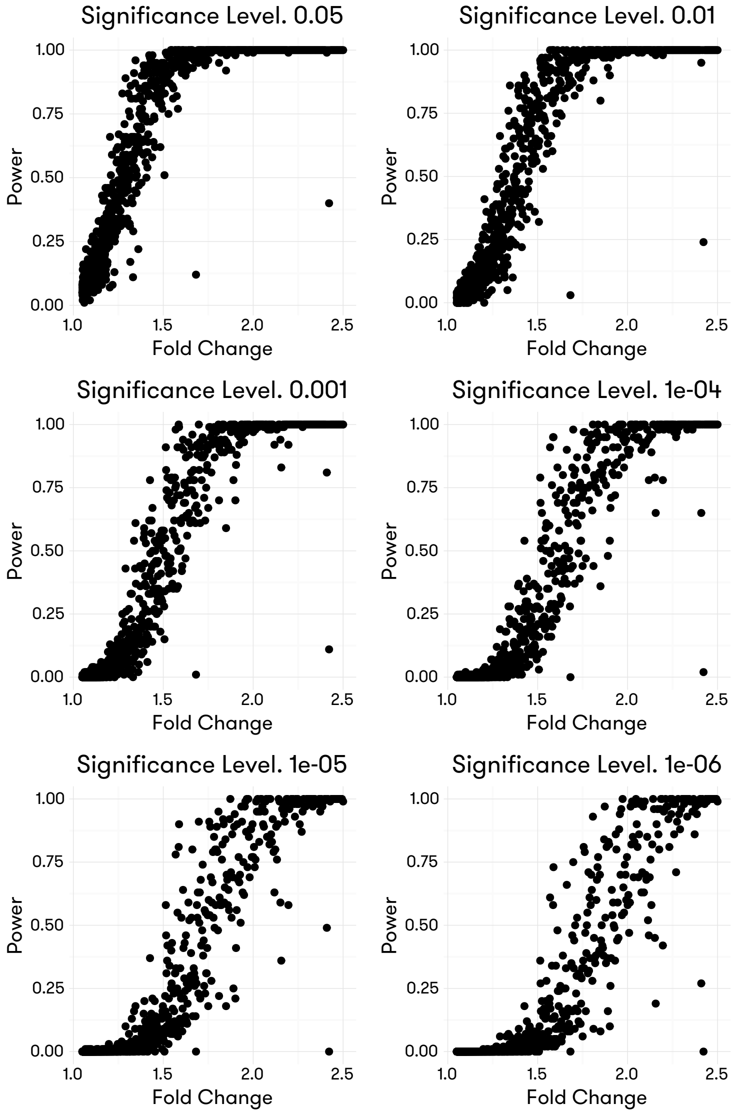

Figure S10: Relationship between the estimated power and the effect size. Each DE gene is plotted with the x axis indicating their η_g. Y axis represents the proportion of datasets in which TASC has called this gene significantly differentially expressed (p is less than or equal to the specified significance level). The sample size of this simulation is 100 vs 100.

SCDE performs quite conservatively in our studies on the type I error. Unsurprisingly, when compared to TASC, has dramatically attenuated power. Figure S11 and Figure S12 illustrate the relationship between the power of and the effect size of gene. With significance levels set at all values (10^{-6} to 0.05) TASC overpowers by a considerable margin. The difference is particularly prominent when the significance level is set below 10^{-4}, which is the common in scRNA-seq analyses due to the preference of controlling for false positives. When the significance level is set to be 10^{-4}, genes with $\eta_g \approx 1.75$ can be detected over 75% of the time by TASC, but less than 25% of the time by SCDE. This is translated into a difference of power between 40% to 80%, a 4-fold improvement in most cases.

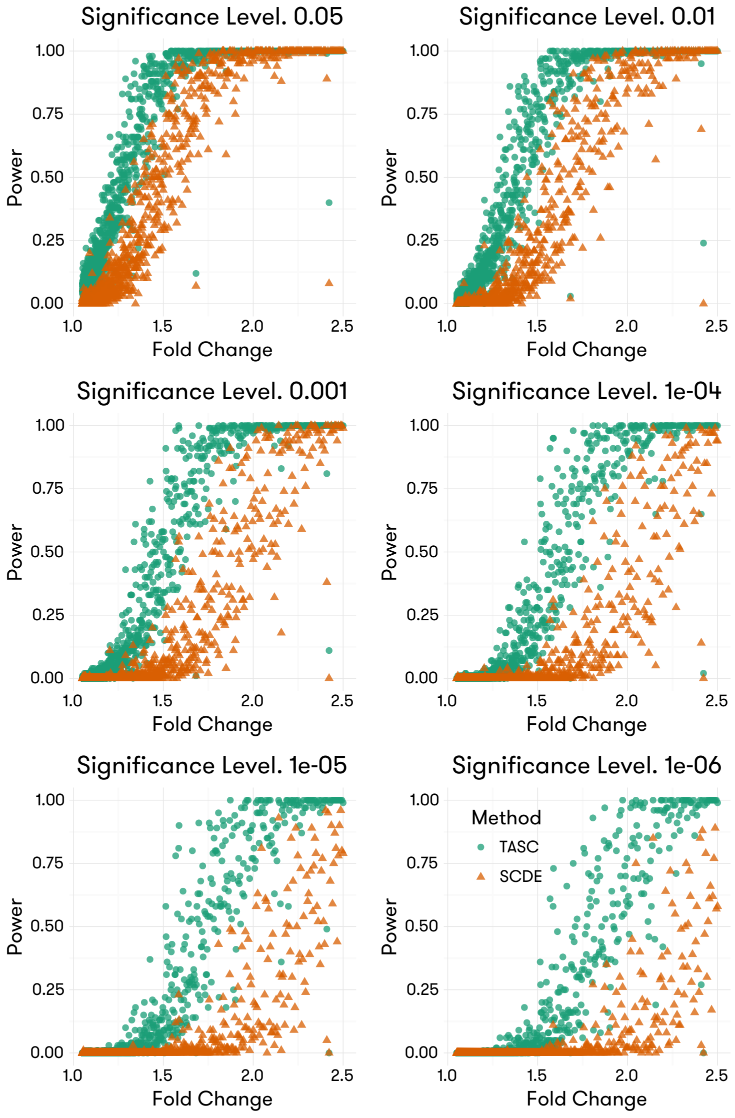

Figure S11: Power comparison between TASC and with various effect sizes. Each panel contains the power curve of TASC and under the specified significance level. This plot is generated from the simulation 100 vs 100 (Figure S7).

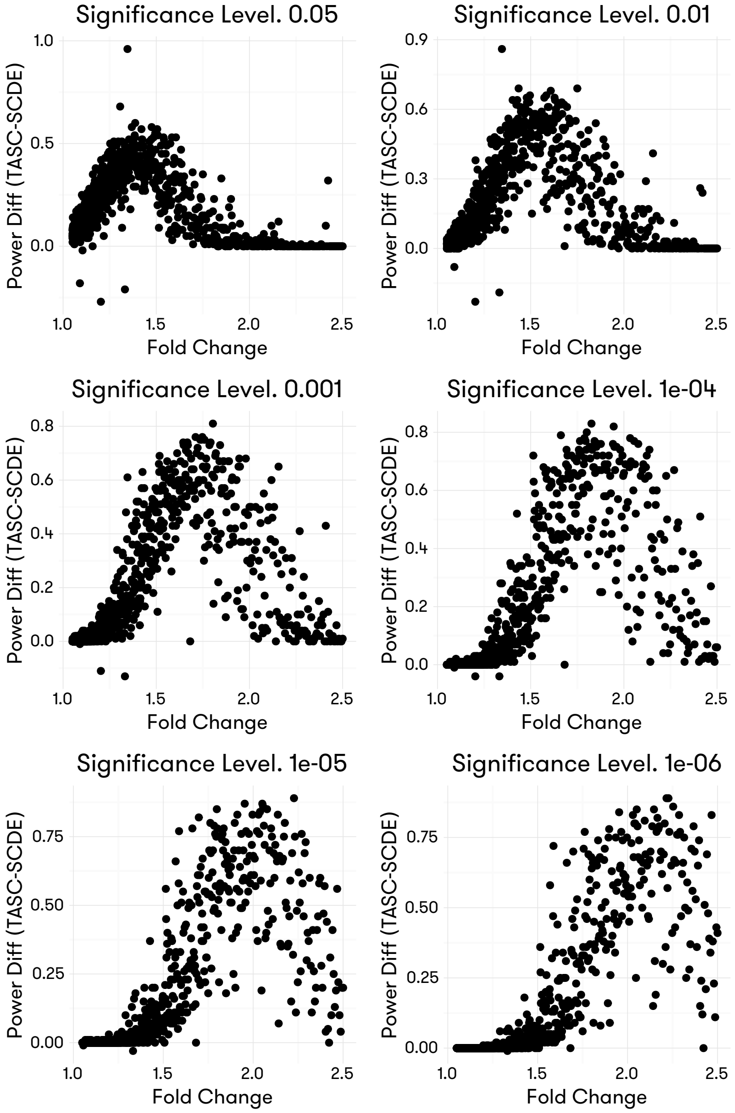

Figure S12: Power improvement of TASC over with various effect sizes. Each panel contains the power improvement curve of TASC and under the specified significance level. Y axis represents the difference in absolute not relative values in estimated power between TASC and , *i.e.* $\omega_g^{TASC} - \omega_g$. This plot is generated from the simulation 100 vs 100 (Figure S7).

Another method specifically designed for scRNA-seq is MAST[5], which shows inflated type I error in our studies based on real data (main text Figures 4A and 4B) even in the absence of batch effects. Among all four methods tested, MAST[5] has the most difficult controlling the type I error rate when batch effects are present in the dataset (main text Figures 5A and 5B). In terms of power, MAST[5] has also performed poorly compared to TASC (Figure S13 and Figure S14). Using genes with $\eta_g \approx 1.75$ as an example, the power difference between TASC and MAST[5] is 10% to over 30%. This suggests that MAST[5] has a tendency to mislabel non-DE genes as DE and DE genes as non-DE, and the results produced by MAST[5] should be validated by other methods to reduce the number of false positives.

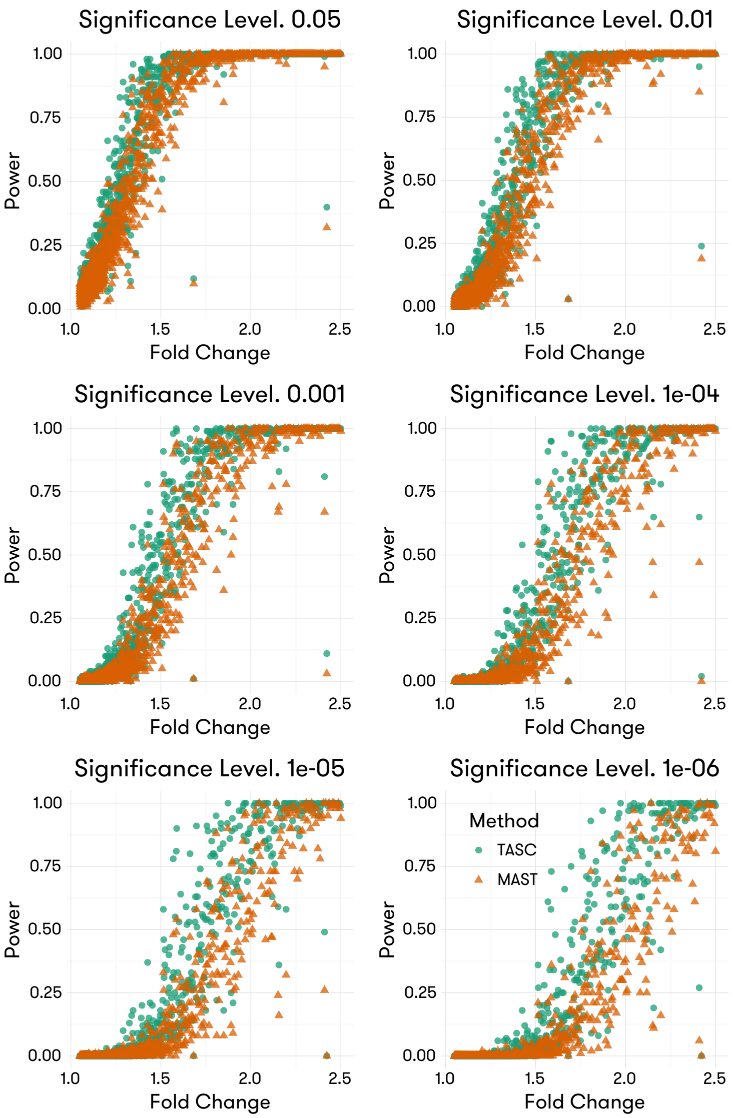

Figure S13: Compare power between TASC and MAST[5] with various effect sizes. Each panel contains the power curve of TASC and MAST[5] under the specified significance level. This plot is generated from the simulation 100 vs 100 (Figure S7).

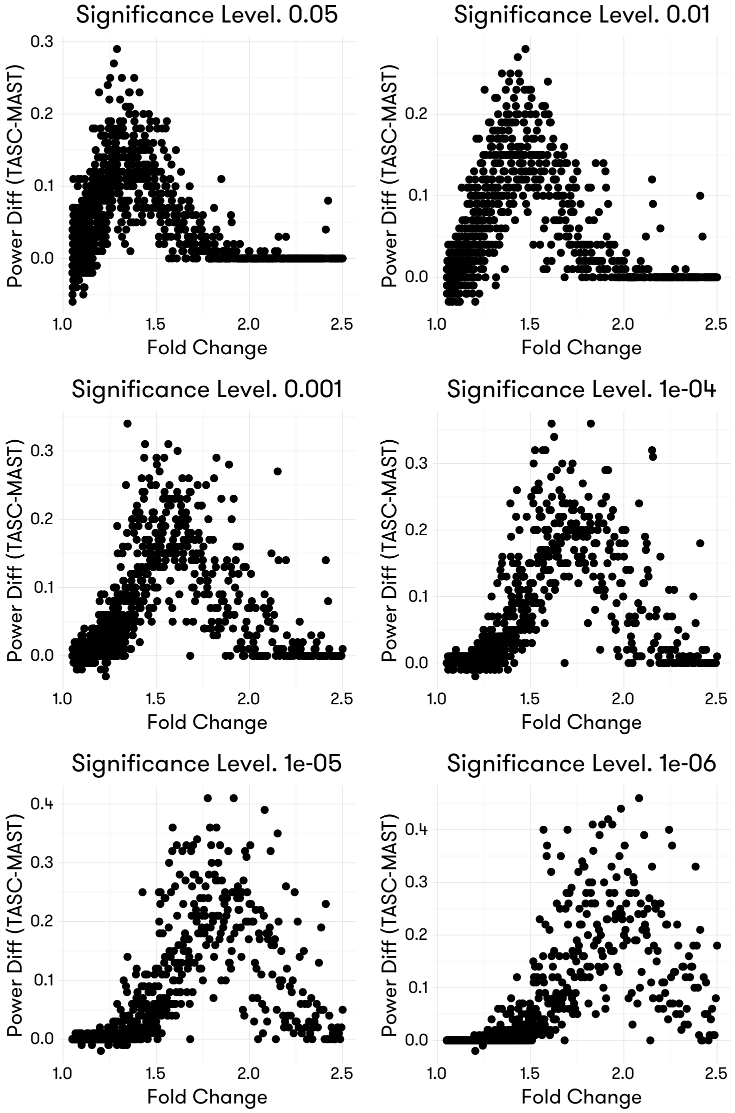

Figure S14: Power improvement of TASC over MAST[5] with various effect sizes. Each panel contains the power improvement curve of TASC and MAST[5] under the specified significance level. Y axis represents the difference in absolute not relative values in estimated power between TASC and MAST[5], *i.e.* $\omega_g^{TASC} - \omega_g^{MAST}$. This plot is generated from the simulation 100 vs 100 (Figure S7).

DESeq2[6] is a popular method for differential expression analysis. Although developed for bulk RNA-seq data, our simulation study suggests that DESeq2 has decent overall performances such as type I error rate (main text Figures 4A and 4B) in the absence of batch effects. In terms of power, however, DESeq2 is outperformed by TASC just like the other methods. Using genes with a fold change near 1.75 as an example, TASC represents a difference of 10% to 30% on the absolute not relative scale over DESeq2. A more troubling issue is that DESeq2[6] lacks the ability to adjust for batch effects and can display serious type I inflation in the presence of batch effects (main text Figures 5A and 5B).

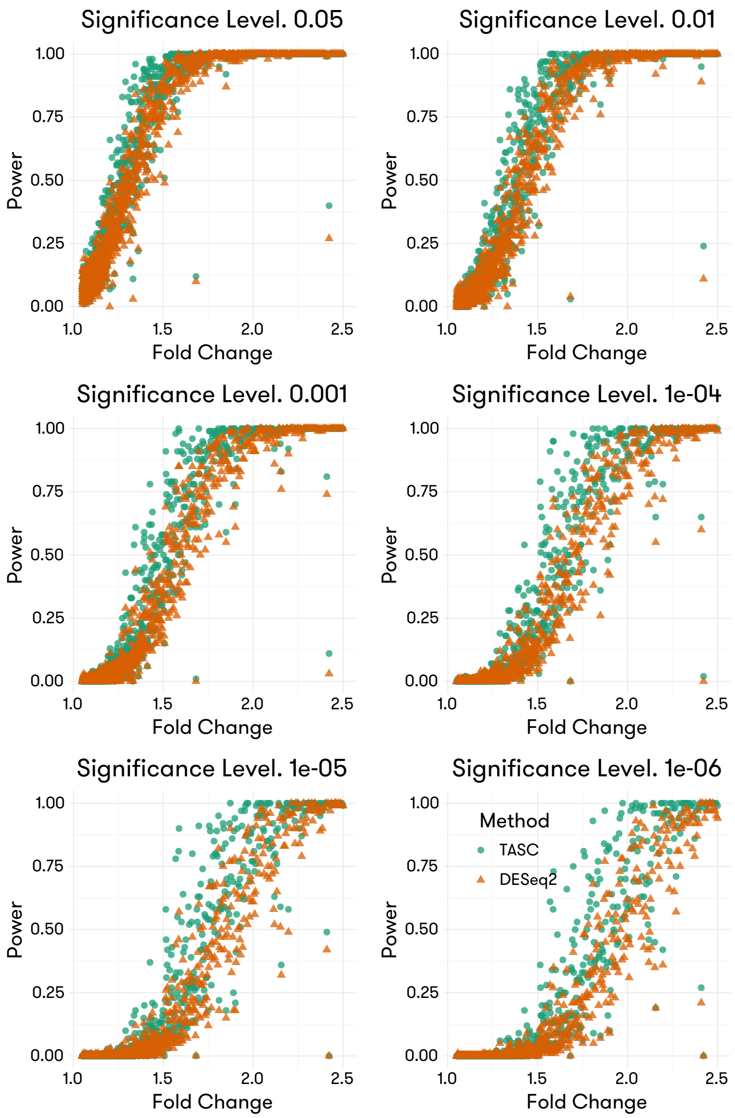

Figure S15: Compare power between TASC and DESeq2[6] with various effect sizes. Each panel contains the power curve of TASC and MAST[5] under the specified significance level. This plot is generated from the simulation 100 vs 100 (Figure S7).

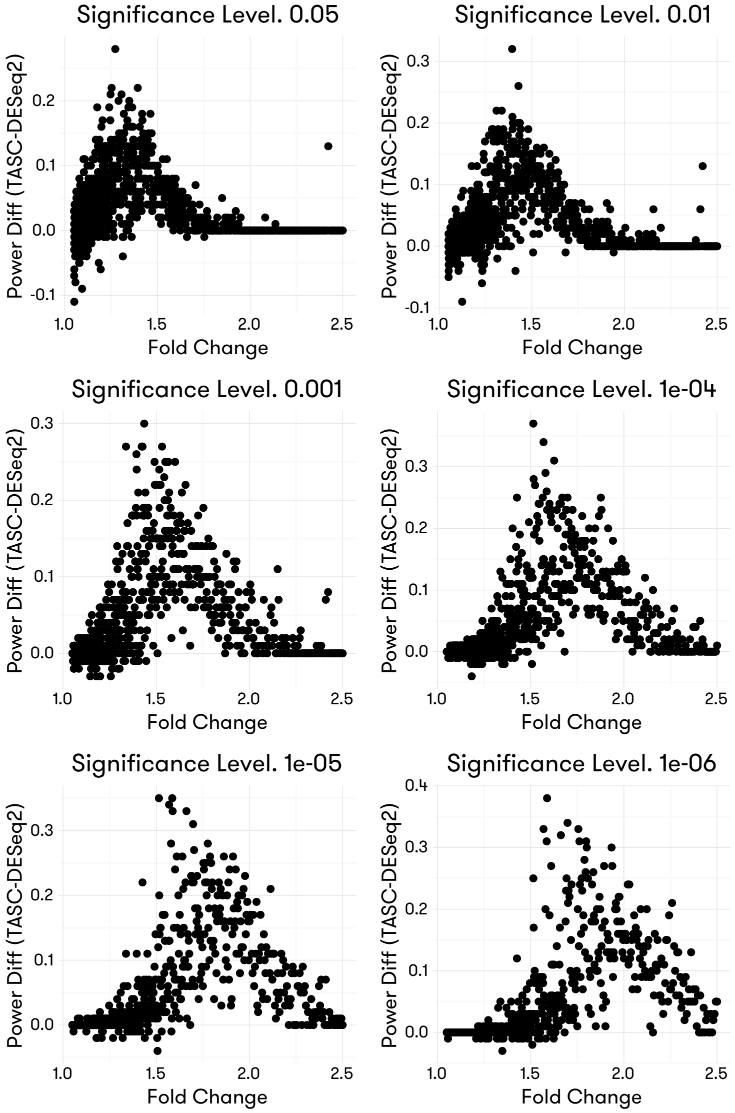

Figure S16: Power improvement of TASC over DESeq2[6] with various effect sizes. Each panel contains the power improvement curve of TASC and DESeq2[6] under the specified significance level. Y axis represents the difference in absolute not relative values in estimated power between TASC and DESeq2[6], *i.e.* $\omega_g^{TASC} - \omega_g^{DESeq2}$. This plot is generated from the simulation 100 vs 100 (Figure S7).

SCRAN[7] is a recently developed method for normalizing scRNA-seq data using cell-specific deconvolved pool-based size factors. As a normalization scheme, its performance is highly dependent on the downstream method of analysis. We have tested SCRAN in the scenario of two-group comparison coupled with DESeq2 and it has shown improved performance over using DESeq2 alone. Since the SCRAN package can also take advantage of the counts for spike-ins to derive the normalization factors, we have looked at both naïve SCRAN (without spike-ins) and SCRAN.SP (SCRAN run with spike-ins). In some cases, due to the limitations of the sample size available, only results from SCRAN.SP are presented.
In terms of power, naïve SCRAN coupled with DESeq2 shows performance similar to DESeq2. In all significance levels tested, TASC overpowers SCRAN+DESeq2 by up to 30%, especially for moderately differentially expressed genes with fold change around 1.75.

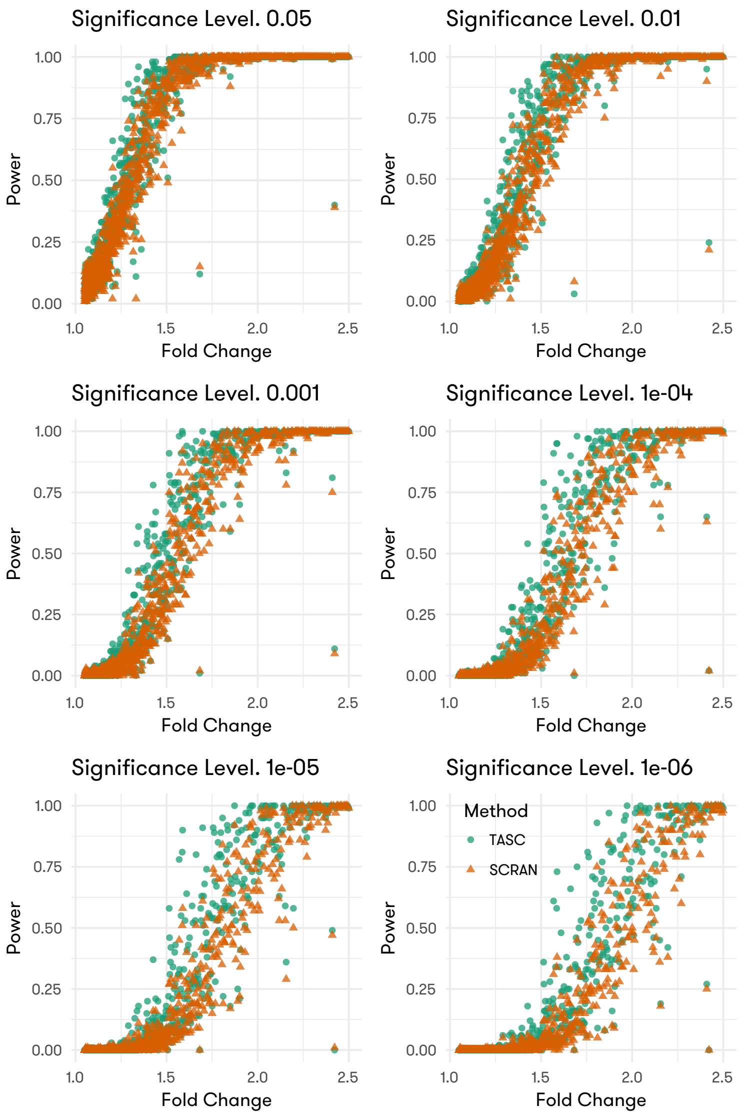

Figure S17: Compare power between TASC and SCRAN[7] with various effect sizes. Each panel contains the power curve of TASC and SCRAN[7] under the specified significance level. This plot is generated from the simulation 100 vs 100 (Figure S7).

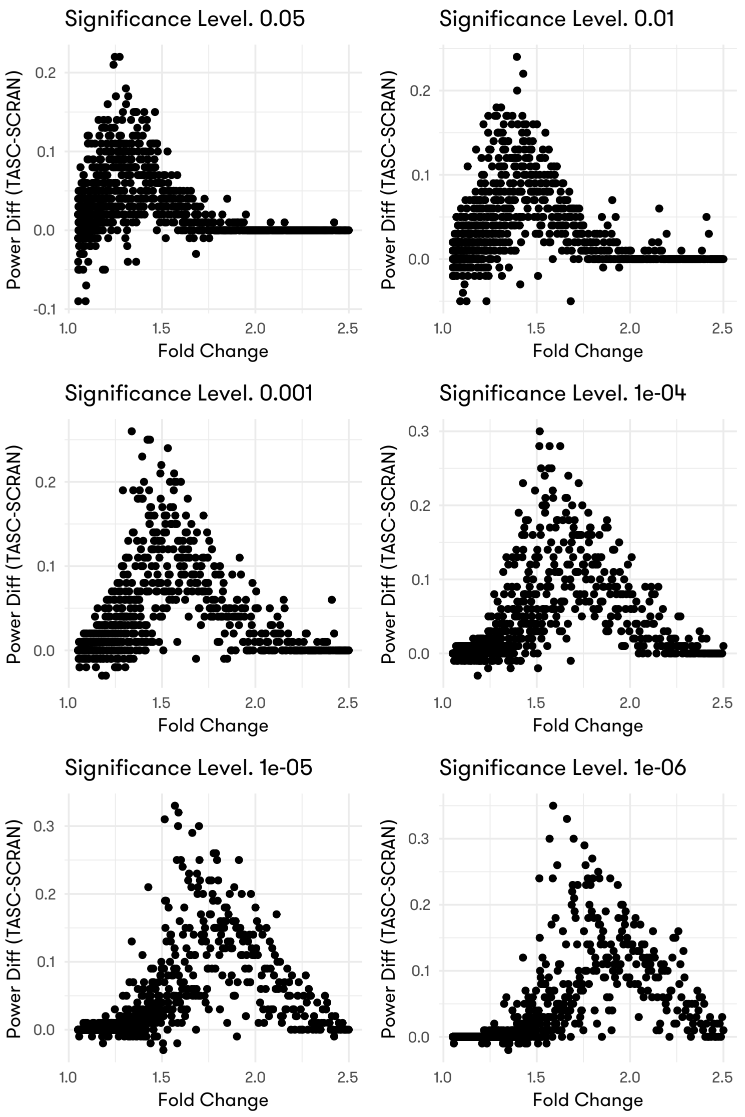

Figure S18: Power improvement of TASC over SCRAN[7] with various effect sizes. Each panel contains the power improvement curve of TASC and SCRAN[7] under the specified significance level. Y axis represents the difference in absolute not relative values in estimated power between TASC and SCRAN[7], *i.e.* $\omega_g^{TASC} - \omega_g^{SCRAN}$. This plot is generated from the simulation 100 vs 100 (Figure S7).

Due to the incorporation of spike-in information, SCRAN.SP coupled with DESeq2 shows profound improvement of power over DESeq2. When compared to TASC, SCRAN.SP is only moderately disadvantaged by up to about 10-20%, the best performer among all methods tested.

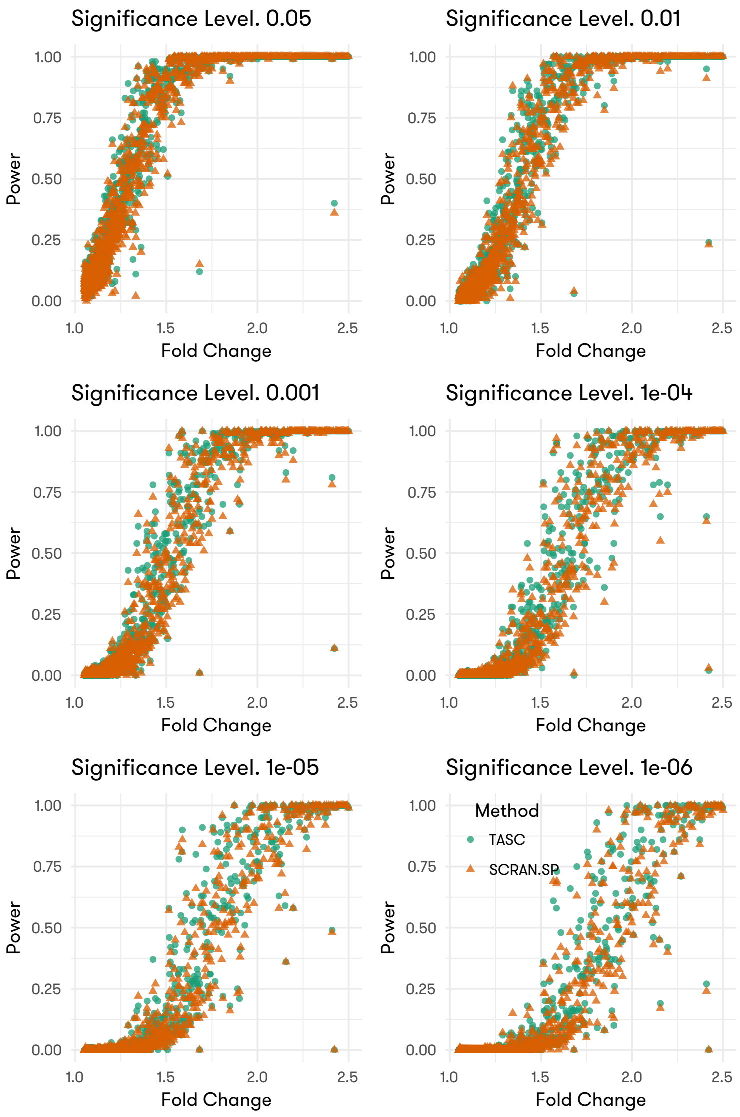

Figure S19: Compare power between TASC and SCRAN[7] with various effect sizes. Each panel contains the power curve of TASC and SCRAN[7] under the specified significance level. This plot is generated from the simulation 100 vs 100 (Figure S7).

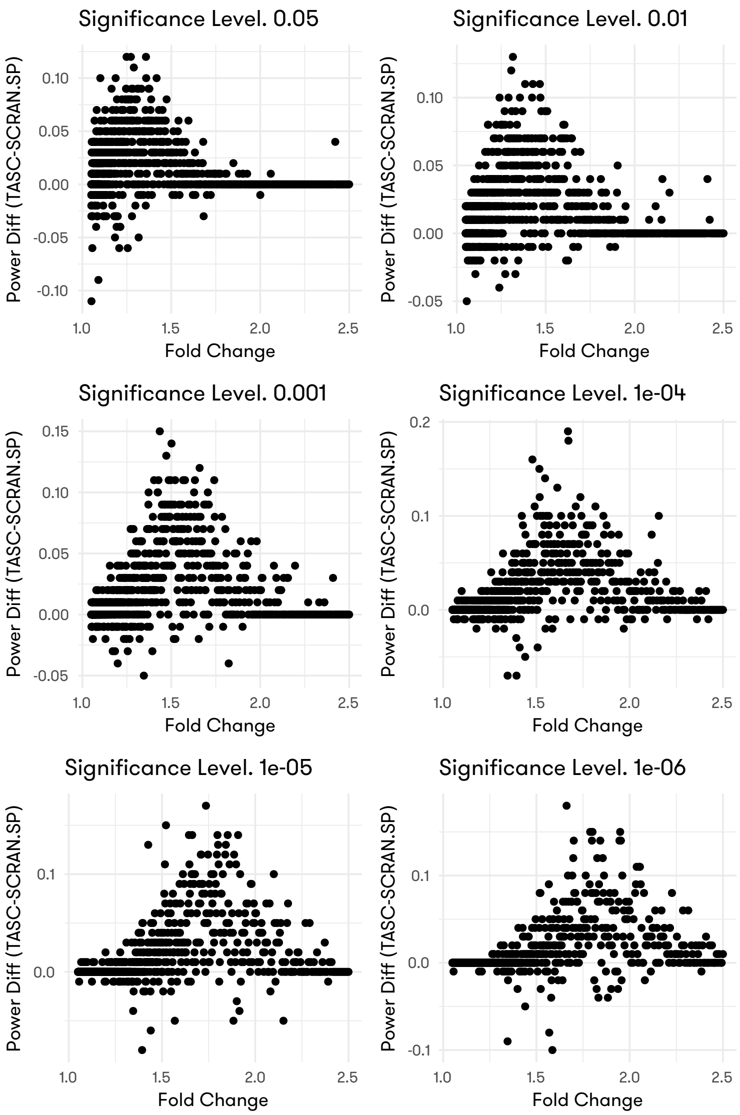

Figure S20: Power improvement of TASC over SCRAN[7] with various effect sizes. Each panel contains the power improvement curve of TASC and SCRAN[7] under the specified significance level. Y axis represents the difference in absolute not relative values in estimated power between TASC and SCRAN[7], *i.e.* $\omega_g^{TASC} - \omega_g^{SCRAN}$. This plot is generated from the simulation 100 vs 100 (Figure S7).

2.3.2 Power and sample size

To investigate the relationship between power achieved by a method and the sample size required, we have down-sampled the complete dataset into varying sizes in different simulations (Figure S7). This has allowed us to look into the behaviour of TASC under different sample size with greater detail.

As the sample size increases, TASC becomes more powerful in detecting small changes in gene expression (Figure S21). When the sample size is only 20 vs 20, TASC has virtually no power except for genes that are highly differentially expressed ($\eta_g \geqslant 2.5$). These genes however can be detected by TASC with almost 100% power when the sample size is equal to or greater than 50 vs 50. For moderately differentially expressed genes ($1.5 < \eta_g < 2$), TASC would require at

least 100 vs 100 to achieve considerable power. For genes with small changes in its expression ($\eta_g < 1.3$), TASC shows no power when the sample size is smaller than or equal to 200 vs 200. However, it is extremely difficult to detect these with significant power without sacrificing the false positive rate.

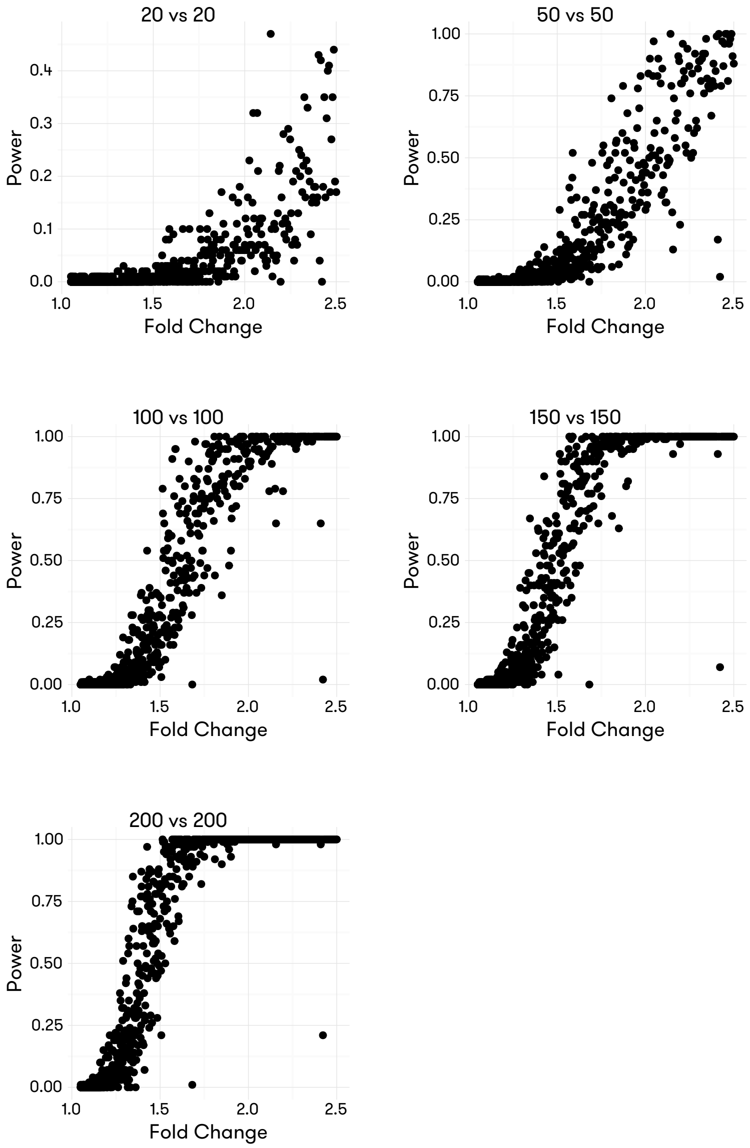

Figure S21: Power curves for TASC from simulations with different sample sizes. In each panel, the estimated power ω_g of each gene for TASC is plotted against the effect size (fold change) assigned for this gene simulated at the specified sample size.

In order to assess the average power for genes of specific effect size, we have used the generalized additive model (GAM) to smooth out the power curve. Briefly, the relationship between estimated power of a gene (ω_g) is regressed onto the fold change assigned to this gene (η_g) using GAM with smooth terms $\mathtt{df} = 4$ and $\mathtt{spar} = 1$ for the spline. Resulting smoothed curves are then plotted for each method under various sample sizes for comparison (main text Figure 6A and S22).

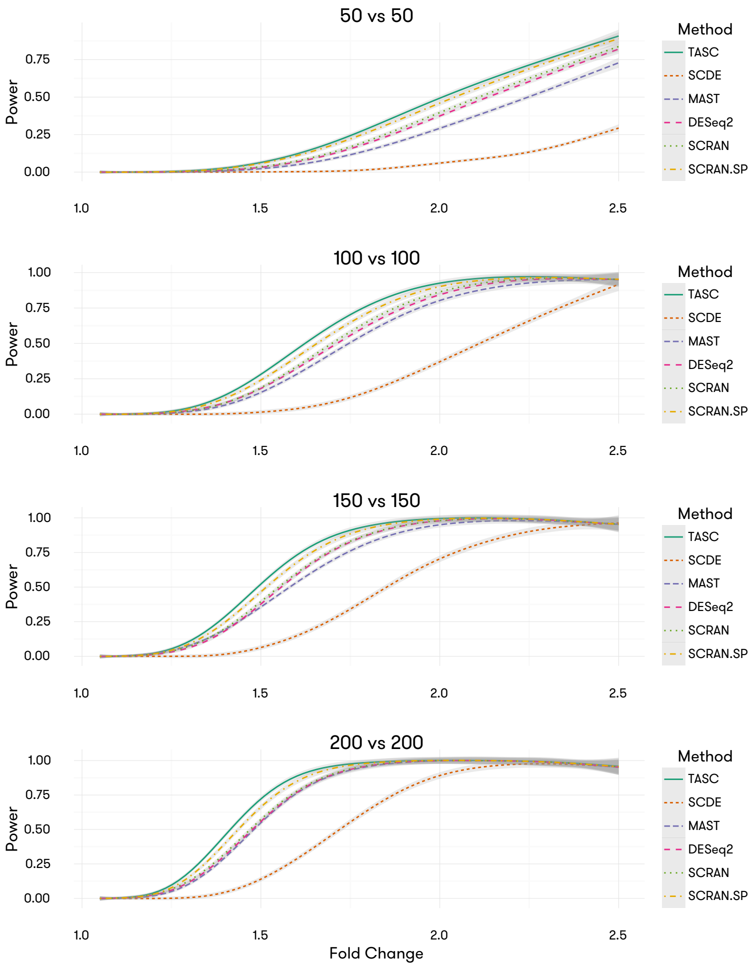

Figure S22: Power curves for TASC, SCDE, MAST, DESeq2, SCRAN and SCRAN.SP for sample sizes of 50 vs 50 and above. In each panel the smoothed power curves for all methods from specified sample size are plotted. X axis indicates the fold change η_g for each gene. Y axis represents the average power for each method after smoothing with GAM as described.

From all simulations of varying sample sizes, TASC has the best power among the four methods tested. TASC is particularly powerful when the genes are only moderately differentially expressed ($\eta \approx 1.75$). This improvement is more dramatic when the sample size is relatively modest (50 vs 50). As the sample size goes up, almost all methods can reliably detect the highly DE genes ($\eta > 2$) with 100% power, which suggests the importance of decently large sample size in single-cell experiments.

2.4 Application to real data

2.4.1 Zeisel et al. data

To gauge the performance of our method in real use case scenarios, we have performed differential gene expression analyses using the two largest level 2 classes of the Zeisel data[1] (“CA1Pyr2” and “CA1Pyr1”). The two groups are downsampled to the indicated sample size in Table S1 and compared using TASC, , MAST[5] and DESeq2[6] respectively. The p-values are extracted from the report of each method and the number of genes that are called significantly is plotted against the size of sub-sample labelled as Table S1.

CA1Pyr2	CA1Pyr1	Numerical Label	Text Label
380	380	380	S32
190	190	190	S16
95	95	95	S8
48	48	48	S4
24	24	24	S2
12	12	12	S1

Table S1: Sample sizes of the sub-sampled Zeisel data[1] sets for two group comparison. Numerical labels are used to approximate the sample sizes in plotting. Text labels are used to distinguish analyses during discussion.

In order to assess the biological relevance of the differentially expressed genes discovered by each method, a gene ontology study has been performed, with results summarized in Tables S2,S3,S4,S5,S6,S7. All genes used are called by each method with p-values smaller than 10^{-8}. This significance level was chosen in order to find the strongest DE genes, while preserving enough genes for meaningful ontology analysis.

2.4.2 SCAP-T data

In order to test the performance of our model using noisier non-UMI data, we have taken advantage of the SCAP-T dataset (description in the main text). This dataset contains the counts of 46,422 endogenous genes and 87 ERCC spike-ins from 198 neurons and 26 astrocytes. Unlike the Zeisel et al data, SCAP-T data is much noisier, and the cells are much more heterogeneous. In Figure S23, a wide range of values for the parameters $(\alpha, \beta, \kappa, \tau)$ can be observed for these samples, and some significant difference exists within the same tissue type as well.

Category	Term	Count	%	PValue	List Total	Pop Hits	Pop Total	Bonferroni	FDR
GOTERM_MF_FAT	GO:0044822 poly(A) RNA binding	319	14.35644	8.13E-59	1802	1197	16866	1.42E-55	1.38E-55
GOTERM_MF_FAT	GO:0003723 RNA binding	400	18.0018	2.47E-58	1802	1707	16866	4.33E-55	4.18E-55
GOTERM_CC_FAT	GO:0098800 inner mitochondrial membrane protein complex	77	3.465347	3.15E-37	1839	124	14413	3.41E-34	5.03E-34
GOTERM_CC_FAT	GO:0098798 mitochondrial protein complex	87	3.915392	1.43E-35	1839	161	14413	1.55E-32	2.28E-32
GOTERM_BP_FAT	GO:0051641 cellular localization	426	19.17192	1.56E-33	1914	2310	17911	1.27E-29	3.13E-30
GOTERM_CC_FAT	GO:0097458 neuron part	371	16.69667	1.88E-33	1839	1620	14413	2.04E-30	3.01E-30
GOTERM_MF_FAT	GO:0003735 structural constituent of ribosome	102	4.590459	1.39E-32	1802	264	16866	2.44E-29	2.36E-29
GOTERM_CC_FAT	GO:0030529 intracellular ribonucleoprotein complex	249	11.20612	2.22E-32	1839	934	14413	2.40E-29	3.54E-29
GOTERM_CC_FAT	GO:1990904 ribonucleoprotein complex	249	11.20612	2.66E-32	1839	935	14413	2.89E-29	4.25E-29
GOTERM_BP_FAT	GO:0043933 macromolecular complex subunit organization	410	18.45185	6.97E-32	1914	2229	17911	5.68E-28	1.39E-28
GOTERM_BP_FAT	GO:0034622 cellular macromolecular complex assembly	211	9.49595	2.19E-30	1914	887	17911	1.78E-26	4.38E-27
GOTERM_CC_FAT	GO:0005840 ribosome	113	5.085509	3.70E-30	1839	286	14413	4.01E-27	5.91E-27
GOTERM_CC_FAT	GO:0044391 ribosomal subunit	95	4.275428	1.16E-29	1839	216	14413	1.26E-26	1.85E-26
GOTERM_BP_FAT	GO:0044085 cellular component biogenesis	459	20.65707	1.51E-29	1914	2654	17911	1.23E-25	3.01E-26
GOTERM_CC_FAT	GO:0044455 mitochondrial membrane part	89	4.005401	1.83E-28	1839	199	14413	1.98E-25	2.92E-25
GOTERM_CC_FAT	GO:0044429 mitochondrial part	228	10.26103	1.97E-28	1839	870	14413	2.13E-25	3.14E-25
GOTERM_CC_FAT	GO:0043005 neuron projection	293	13.18632	1.41E-27	1839	1253	14413	1.53E-24	2.25E-24
GOTERM_CC_FAT	GO:0005743 mitochondrial inner membrane	137	6.165617	3.13E-27	1839	415	14413	3.39E-24	5.00E-24
GOTERM_BP_FAT	GO:0022607 cellular component assembly	419	18.85689	7.11E-27	1914	2418	17911	5.79E-23	1.42E-23

Table S2: Top 20 GO terms discovered for differentially expressed genes called by TASC.

Category	Term	Count	%	PValue	List Total	Pop Hits	Pop Total	Bonferroni	FDR
GOTERM_CC_FAT	GO:0098800 inner mitochondrial membrane protein complex	72	5.930807	3.52E-49	1027	124	14413	3.13E-46	5.48E-46
GOTERM_CC_FAT	GO:0098798 mitochondrial protein complex	81	6.672158	5.84E-49	1027	161	14413	5.21E-46	9.10E-46
GOTERM_CC_FAT	GO:0044455 mitochondrial membrane part	82	6.75453	2.42E-41	1027	199	14413	2.16E-38	3.77E-38
GOTERM_CC_FAT	GO:0070469 respiratory chain	53	4.365733	3.06E-37	1027	88	14413	2.72E-34	4.76E-34
GOTERM_CC_FAT	GO:0005746 mitochondrial respiratory chain	50	4.118616	3.19E-36	1027	80	14413	2.84E-33	4.96E-33
GOTERM_CC_FAT	GO:0044429 mitochondrial part	170	14.00329	1.92E-35	1027	870	14413	1.71E-32	2.99E-32
GOTERM_CC_FAT	GO:0005743 mitochondrial inner membrane	111	9.143328	2.08E-35	1027	415	14413	1.86E-32	3.24E-32
GOTERM_CC_FAT	GO:0005740 mitochondrial envelope	143	11.77924	2.79E-35	1027	652	14413	2.49E-32	4.35E-32
GOTERM_CC_FAT	GO:0098803 respiratory chain complex	48	3.953871	5.85E-34	1027	79	14413	5.21E-31	9.11E-31
GOTERM_CC_FAT	GO:0031966 mitochondrial membrane	135	11.12026	6.40E-34	1027	607	14413	5.70E-31	9.96E-31
GOTERM_CC_FAT	GO:0019866 organelle inner membrane	112	9.2257	4.20E-31	1027	467	14413	3.74E-28	6.54E-28
GOTERM_CC_FAT	GO:0005739 mitochondrion	254	20.92257	2.18E-29	1027	1792	14413	1.94E-26	3.39E-26
GOTERM_CC_FAT	GO:0031967 organelle envelope	178	14.66227	3.89E-28	1027	1069	14413	3.46E-25	6.05E-25
GOTERM_CC_FAT	GO:0031975 envelope	178	14.66227	6.90E-28	1027	1074	14413	6.14E-25	1.07E-24
GOTERM_CC_FAT	GO:0030964 NADH dehydrogenase complex	34	2.800659	1.04E-27	1027	47	14413	9.27E-25	1.62E-24
GOTERM_CC_FAT	GO:0045271 respiratory chain complex I	34	2.800659	1.04E-27	1027	47	14413	9.27E-25	1.62E-24
GOTERM_CC_FAT	GO:0005747 mitochondrial respiratory chain complex I	34	2.800659	1.04E-27	1027	47	14413	9.27E-25	1.62E-24
GOTERM_CC_FAT	GO:0097458 neuron part	233	19.19275	1.22E-27	1027	1620	14413	1.09E-24	1.90E-24
GOTERM_CC_FAT	GO:0043005 neuron projection	193	15.89786	3.11E-26	1027	1253	14413	2.77E-23	4.85E-23

Table S3: Top 20 GO terms discovered for differentially expressed genes called by SCDE.

Category	Term	Count	%	PValue	List Total	Pop Hits	Pop Total	Bonferroni	FDR
GOTERM_MF_FAT	GO:0044822 poly(A) RNA binding	301	13.87097	1.51E-51	1763	1197	16866	2.61E-48	2.55E-48
GOTERM_MF_FAT	GO:0003723 RNA binding	376	17.32719	7.54E-50	1763	1707	16866	1.30E-46	1.27E-46
GOTERM_CC_FAT	GO:0098798 mitochondrial protein complex	88	4.0553	2.26E-37	1792	161	14413	2.43E-34	3.61E-34
GOTERM_CC_FAT	GO:0098800 inner mitochondrial membrane protein complex	76	3.502304	5.74E-37	1792	124	14413	6.15E-34	9.14E-34
GOTERM_CC_FAT	GO:0097458 neuron part	368	16.95853	7.42E-35	1792	1620	14413	7.96E-32	1.18E-31
GOTERM_BP_FAT	GO:0051641 cellular localization	418	19.26267	8.36E-33	1879	2310	17911	6.72E-29	1.67E-29
GOTERM_BP_FAT	GO:0043933 macromolecular complex subunit organization	404	18.61751	9.54E-32	1879	2229	17911	7.66E-28	1.91E-28
GOTERM_CC_FAT	GO:0044455 mitochondrial membrane part	90	4.147465	4.56E-30	1792	199	14413	4.89E-27	7.26E-27
GOTERM_BP_FAT	GO:0034622 cellular macromolecular complex assembly	207	9.539171	1.03E-29	1879	887	17911	8.29E-26	2.06E-26
GOTERM_CC_FAT	GO:0030529 intracellular ribonucleoprotein complex	238	10.96774	1.75E-29	1792	934	14413	1.88E-26	2.79E-26
GOTERM_CC_FAT	GO:1990904 ribonucleoprotein complex	238	10.96774	2.07E-29	1792	935	14413	2.22E-26	3.30E-26
GOTERM_CC_FAT	GO:0043005 neuron projection	289	13.31797	4.62E-28	1792	1253	14413	4.96E-25	7.37E-25
GOTERM_BP_FAT	GO:0044085 cellular component biogenesis	447	20.59908	6.43E-28	1879	2654	17911	5.16E-24	1.28E-24
GOTERM_CC_FAT	GO:0070469 respiratory chain	55	2.534562	2.07E-27	1792	88	14413	2.22E-24	3.30E-24
GOTERM_CC_FAT	GO:0005746 mitochondrial respiratory chain	52	2.396313	4.24E-27	1792	80	14413	4.55E-24	6.75E-24
GOTERM_MF_FAT	GO:0003735 structural constituent of ribosome	93	4.285714	5.82E-27	1763	264	16866	1.01E-23	9.83E-24
GOTERM_CC_FAT	GO:0044429 mitochondrial part	220	10.13825	1.00E-26	1792	870	14413	1.08E-23	1.60E-23
GOTERM_CC_FAT	GO:0005743 mitochondrial inner membrane	133	6.129032	3.69E-26	1792	415	14413	3.96E-23	5.89E-23
GOTERM_CC_FAT	GO:0044456 synapse part	187	8.617512	6.23E-26	1792	697	14413	6.69E-23	9.94E-23

Table S4: Top 20 GO terms discovered for differentially expressed genes called by MAST.

Category	Term	Count	%	PValue	List Total	Pop Hits	Pop Total	Bonferroni	FDR
GOTERM_MF_FAT	GO:0003723 RNA binding	403	18.16133	2.82E-62	1767	1707	16866	4.91E-59	4.77E-59
GOTERM_MF_FAT	GO:0044822 poly(A) RNA binding	320	14.42091	1.93E-61	1767	1197	16866	3.37E-58	3.27E-58
GOTERM_CC_FAT	GO:0030529 intracellular ribonucleoprotein complex	274	12.3479	3.36E-44	1835	934	14413	3.64E-41	5.36E-41
GOTERM_CC_FAT	GO:1990904 ribonucleoprotein complex	274	12.3479	4.19E-44	1835	935	14413	4.55E-41	6.70E-41
GOTERM_CC_FAT	GO:0098800 inner mitochondrial membrane protein complex	80	3.605228	1.63E-40	1835	124	14413	1.77E-37	2.60E-37
GOTERM_MF_FAT	GO:0003735 structural constituent of ribosome	110	4.957188	1.69E-39	1767	264	16866	2.94E-36	2.86E-36
GOTERM_CC_FAT	GO:0005840 ribosome	126	5.678233	1.82E-39	1835	286	14413	1.98E-36	2.91E-36
GOTERM_CC_FAT	GO:0098798 mitochondrial protein complex	90	4.055881	1.96E-38	1835	161	14413	2.12E-35	3.13E-35
GOTERM_CC_FAT	GO:0044391 ribosomal subunit	103	4.641731	6.10E-36	1835	216	14413	6.62E-33	9.73E-33
GOTERM_CC_FAT	GO:0044429 mitochondrial part	244	10.99594	1.21E-35	1835	870	14413	1.32E-32	1.94E-32
GOTERM_CC_FAT	GO:0044455 mitochondrial membrane part	96	4.326273	4.99E-34	1835	199	14413	5.41E-31	7.96E-31
GOTERM_CC_FAT	GO:0005739 mitochondrion	400	18.02614	6.55E-34	1835	1792	14413	7.10E-31	1.04E-30
GOTERM_BP_FAT	GO:0051641 cellular localization	423	19.06264	6.31E-33	1907	2310	17911	5.08E-29	1.26E-29
GOTERM_BP_FAT	GO:0006518 peptide metabolic process	209	9.418657	8.62E-31	1907	872	17911	6.94E-27	1.72E-27
GOTERM_BP_FAT	GO:0034622 cellular macromolecular complex assembly	211	9.508788	1.31E-30	1907	887	17911	1.06E-26	2.62E-27
GOTERM_BP_FAT	GO:0006412 translation	182	8.201893	2.91E-30	1907	714	17911	2.34E-26	5.81E-27
GOTERM_CC_FAT	GO:0005743 mitochondrial inner membrane	142	6.399279	3.94E-30	1835	415	14413	4.27E-27	6.29E-27
GOTERM_BP_FAT	GO:0043043 peptide biosynthetic process	185	8.337089	5.04E-30	1907	735	17911	4.05E-26	1.01E-26
GOTERM_BP_FAT	GO:0044085 cellular component biogenesis	459	20.68499	6.21E-30	1907	2654	17911	5.00E-26	1.24E-26

Table S5: Top 20 GO terms discovered for differentially expressed genes called by DESeq2.

Category	Term	Count	%	PValue	List Total	Pop Hits	Pop Total	Bonferroni	FDR
GOTERM_MF_FAT	GO:0003723 RNA binding	386	18.60241	9.88E-62	1660	1707	16866	1.67E-58	1.66E-58
GOTERM_MF_FAT	GO:0044822 poly(A) RNA binding	308	14.84337	2.49E-61	1660	1197	16866	4.22E-58	4.20E-58
GOTERM_CC_FAT	GO:0030529 intracellular ribonucleoprotein complex	268	12.91566	2.49E-46	1727	934	14413	2.64E-43	3.96E-43
GOTERM_CC_FAT	GO:1990904 ribonucleoprotein complex	268	12.91566	3.10E-46	1727	935	14413	3.30E-43	4.94E-43
GOTERM_CC_FAT	GO:0098800 inner mitochondrial membrane protein complex	80	3.855422	1.78E-42	1727	124	14413	1.89E-39	2.83E-39
GOTERM_CC_FAT	GO:0005840 ribosome	125	6.024096	1.69E-41	1727	286	14413	1.79E-38	2.69E-38
GOTERM_CC_FAT	GO:0098798 mitochondrial protein complex	90	4.337349	1.43E-40	1727	161	14413	1.51E-37	2.27E-37
GOTERM_MF_FAT	GO:0003735 structural constituent of ribosome	108	5.204819	2.02E-40	1660	264	16866	3.42E-37	3.40E-37
GOTERM_CC_FAT	GO:0044429 mitochondrial part	243	11.71084	1.21E-39	1727	870	14413	1.29E-36	1.93E-36
GOTERM_CC_FAT	GO:0044391 ribosomal subunit	102	4.915663	1.90E-37	1727	216	14413	2.02E-34	3.03E-34
GOTERM_CC_FAT	GO:0005739 mitochondrion	391	18.84337	6.36E-37	1727	1792	14413	6.76E-34	1.01E-33
GOTERM_CC_FAT	GO:0044455 mitochondrial membrane part	96	4.626506	3.26E-36	1727	199	14413	3.46E-33	5.19E-33
GOTERM_CC_FAT	GO:0005743 mitochondrial inner membrane	141	6.795181	2.27E-32	1727	415	14413	2.41E-29	3.62E-29
GOTERM_BP_FAT	GO:0006518 peptide metabolic process	203	9.783133	1.50E-31	1799	872	17911	1.18E-27	3.00E-28
GOTERM_CC_FAT	GO:0005740 mitochondrial envelope	186	8.963855	2.10E-31	1727	652	14413	2.23E-28	3.35E-28
GOTERM_BP_FAT	GO:0034622 cellular macromolecular complex assembly	203	9.783133	1.72E-30	1799	887	17911	1.35E-26	3.43E-27
GOTERM_BP_FAT	GO:0006412 translation	176	8.481928	1.79E-30	1799	714	17911	1.41E-26	3.56E-27
GOTERM_BP_FAT	GO:0043043 peptide biosynthetic process	179	8.626506	2.63E-30	1799	735	17911	2.07E-26	5.25E-27
GOTERM_BP_FAT	GO:0051641 cellular localization	394	18.98795	2.57E-29	1799	2310	17911	2.02E-25	5.12E-26

Table S6: Top 20 GO terms discovered for differentially expressed genes called by SCRAN.

Category	Term	Count	%	PValue	List Total	Pop Hits	Pop Total	Bonferroni	FDR
GOTERM_CC_FAT	GO:0097458 neuron part	496	18.30934	1.42E-61	2217	1620	14413	1.62E-58	2.28E-58
GOTERM_BP_FAT	GO:0051641 cellular localization	561	20.70875	3.69E-55	2363	2310	17911	3.26E-51	7.44E-52
GOTERM_MF_FAT	GO:0044822 poly(A) RNA binding	355	13.10447	1.81E-54	2229	1197	16866	3.51E-51	3.10E-51
GOTERM_CC_FAT	GO:0043005 neuron projection	395	14.58103	1.86E-51	2217	1253	14413	2.12E-48	2.98E-48
GOTERM_BP_FAT	GO:0007399 nervous system development	540	19.93355	2.37E-50	2363	2261	17911	2.09E-46	4.77E-47
GOTERM_CC_FAT	GO:0044456 synapse part	264	9.745293	6.06E-50	2217	697	14413	6.91E-47	9.74E-47
GOTERM_BP_FAT	GO:0031175 neuron projection development	289	10.66814	3.41E-49	2363	921	17911	3.01E-45	6.87E-46
GOTERM_MF_FAT	GO:0003723 RNA binding	438	16.16833	1.04E-48	2229	1707	16866	2.01E-45	1.78E-45
GOTERM_CC_FAT	GO:0045202 synapse	304	11.22185	2.01E-48	2217	876	14413	2.29E-45	3.23E-45
GOTERM_BP_FAT	GO:0048666 neuron development	318	11.73865	1.54E-46	2363	1092	17911	1.36E-42	3.11E-43
GOTERM_BP_FAT	GO:0030182 neuron differentiation	367	13.54743	5.47E-44	2363	1379	17911	4.83E-40	1.10E-40
GOTERM_BP_FAT	GO:0048699 generation of neurons	394	14.54411	5.48E-44	2363	1526	17911	4.84E-40	1.10E-40
GOTERM_BP_FAT	GO:0051649 establishment of localization in cell	429	15.8361	6.74E-44	2363	1722	17911	5.95E-40	1.36E-40
GOTERM_CC_FAT	GO:0036477 somatodendritic compartment	301	11.11111	5.80E-42	2217	921	14413	6.62E-39	9.32E-39
GOTERM_BP_FAT	GO:0022008 neurogenesis	409	15.09782	6.04E-42	2363	1638	17911	5.34E-38	1.22E-38
GOTERM_BP_FAT	GO:0051128 regulation of cellular component organization	536	19.7859	1.07E-40	2363	2399	17911	9.41E-37	2.15E-37
GOTERM_BP_FAT	GO:0048812 neuron projection morphogenesis	196	7.235142	2.51E-39	2363	570	17911	2.21E-35	5.05E-36
GOTERM_BP_FAT	GO:0043933 macromolecular complex subunit organization	501	18.49391	1.83E-38	2363	2229	17911	1.61E-34	3.68E-35
GOTERM_BP_FAT	GO:0046907 intracellular transport	337	12.44001	2.28E-37	2363	1304	17911	2.02E-33	4.61E-34

Table S7: Top 20 GO terms discovered for differentially expressed genes called by SCRAN.SP.

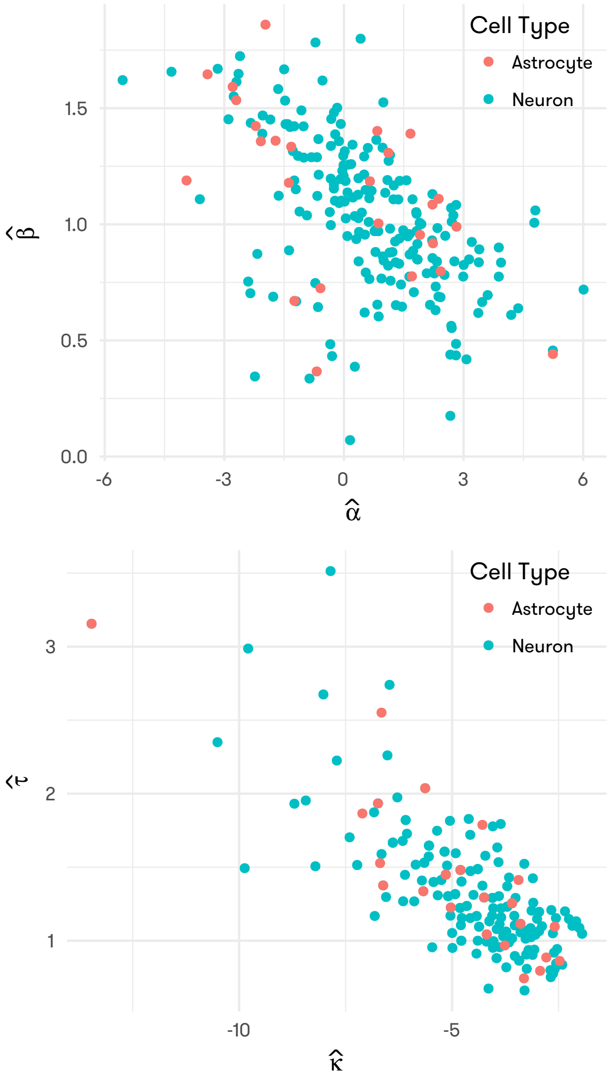

Figure S23: Scatter plots describing the distribution of $\boldsymbol{\Psi}_c$ of the SCAP-T data.

Before we can use ERCC spike-ins in the SCAP-T data to model the technical noise, necessary pre-processing is required to tease out the cells that are of low quality. One can achieve this by looking at the R^2 values from the linear regression with the log counts as the response variable, and the log true concentration of the ERCC as the input covariate. SCAP-T data obviously has much wider range of R^2 (Figure S24) compared to Zeisel et al. data (Figure S27), suggesting some trimming might be necessary to remove those cells with really low R^2 if TASC is to be used.

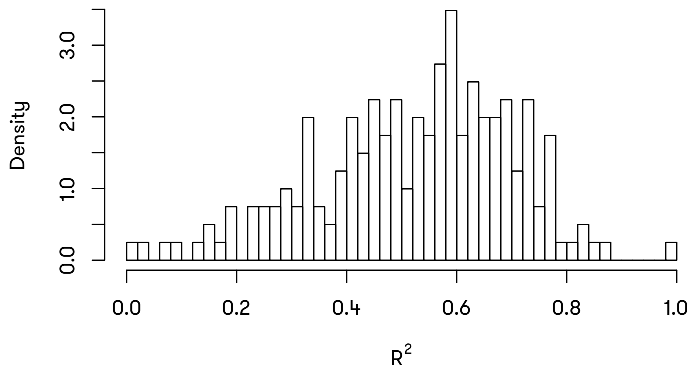

Figure S24: Histograms for R^2 computed from SCAP-T data.

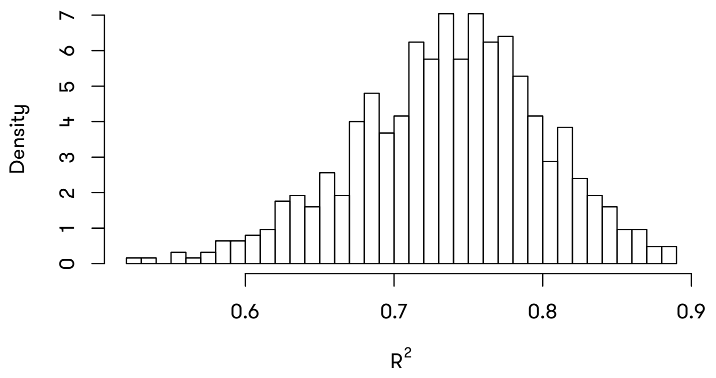

Figure S25: Histograms for R^2 computed from Zeisel et al. data.

Another characteristic of the SCAP-T data is the more varied sample size. We have plotted the normalized cell size factors computed from SCAP-T (Figure S26) and Zeisel et al. (Figure S27) data. It is obvious that the former has much wider range of cell size factors, which indicate that some of the cells in this data set might contain too many or too few reads coming from the biological genes, both of which will affect the accuracy of TASC.

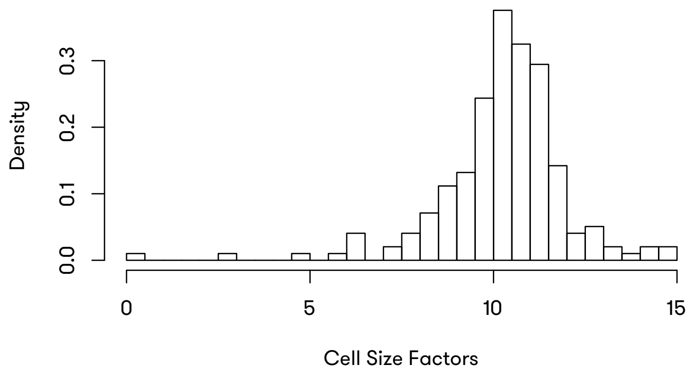

Figure S26: Histograms for normalized cell size factors computed from SCAP-T data.

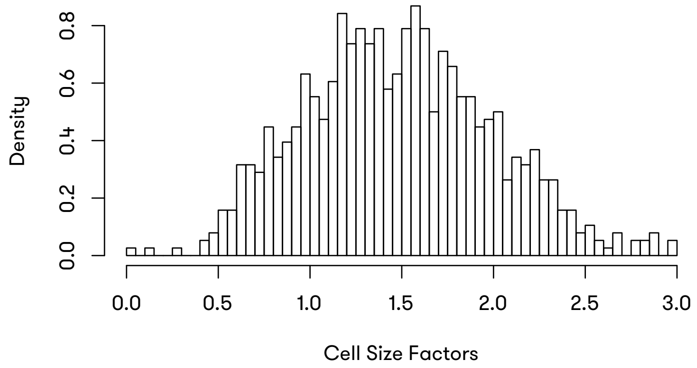

Figure S27: Histograms for normalized cell size factors computed from Zeisel et al. data.

In order to assess the type I error rates of the tested methods for non-UMI datasets in the presence of varying technical noise. We have derived a null comparison following these steps.

Step.*a* Estimate the technical parameters $(\alpha, \beta, \kappa, \tau)$ for both astrocytes and neurons.

Step.*b* Find those neurons with the technical parameters closest in Euclidean distance to that of each astrocyte, and label these neurons as group 1. If multiple astrocytes share one closest neuron, then multiple neurons are selected for these astrocytes.

Step.*c* Label the unchosen neurons as group 2.

Differential gene expression analyses have been performed on these two groups with all five methods (naïve SCRAN is not available due to the small sample size in group 1). Raw p-values are plotted using histograms (Figure S28). Negative logarithm of the raw p-values with base 10 are plotted with Q-Q plots (Figure S29).
From both plots, under the null condition, the p-values from TASC are uniformly distributed, with no inflation. However, p-values from all the other methods both show distinct signs of

being overly conservative as well as inflated type I error rates. This is consistent with our simulation results, *i.e.*, in the presence of batch effects, only TASC has type I error rate under control.

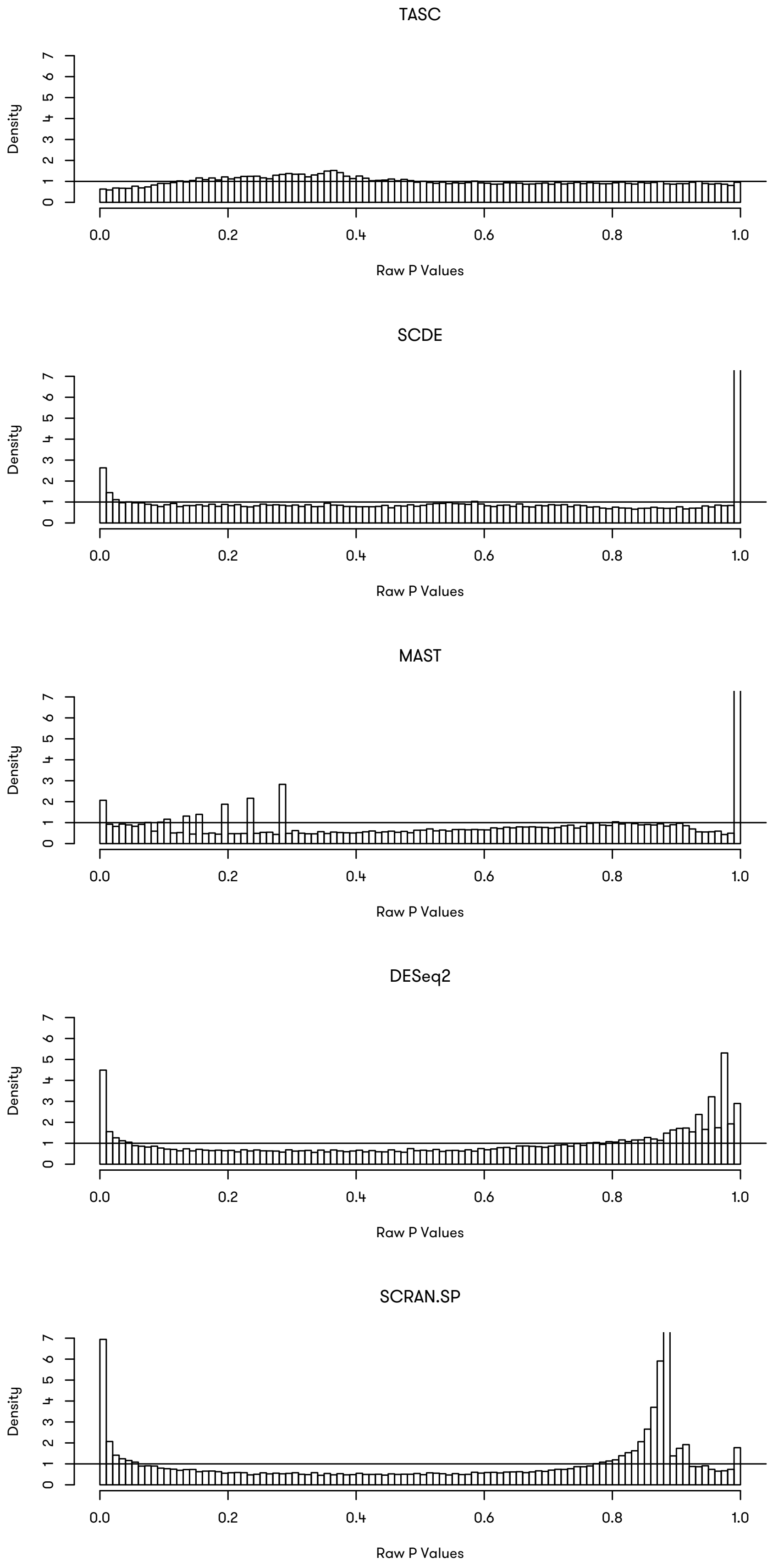

Figure S28: Histograms describing the distributions of raw p-values from various methods in the null comparison with SCAP-T data.

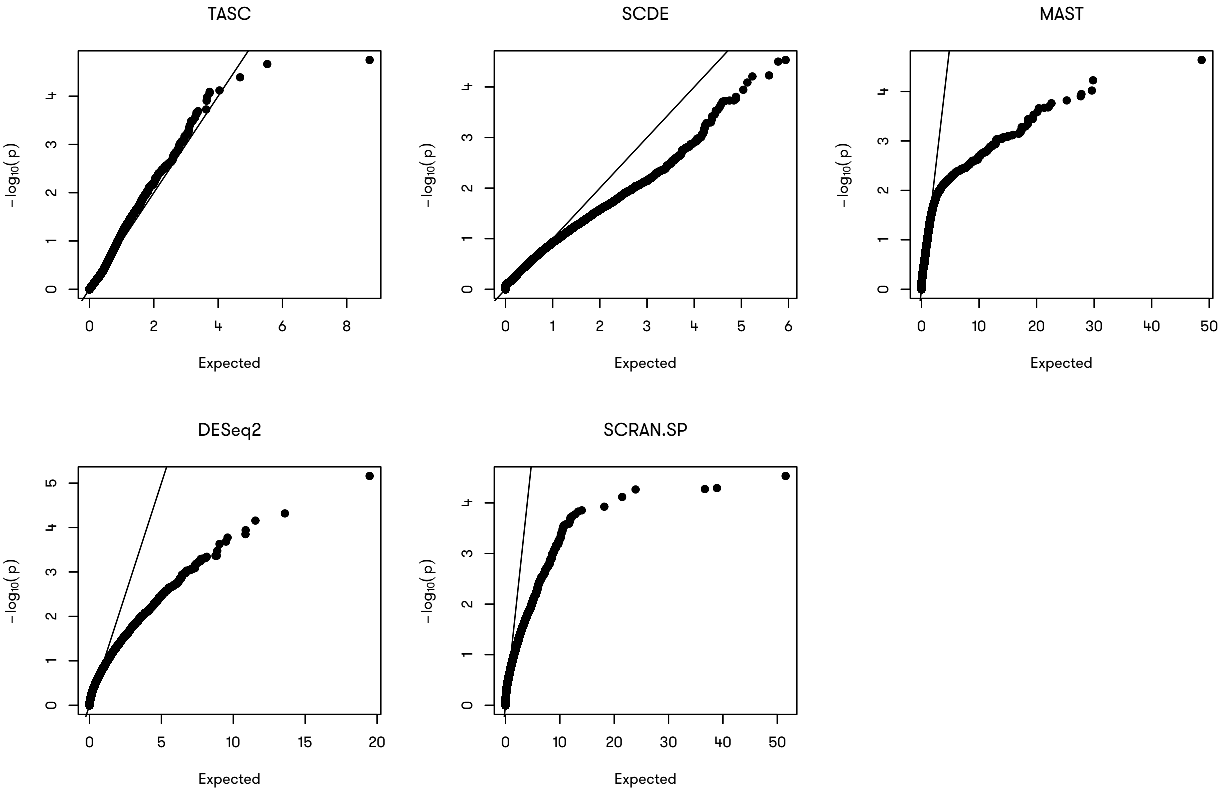

Figure S29: Q-Q plots describing the distributions of raw p-values from various methods in the null comparison with SCAP-T data.

3 Computational Details

3.1 Laplace Approximation

In order to speed up the evaluation of integral, we have adopted Laplace's method to approximate the value and reduce the required computational resources. Briefly, the marginal likelihood of one cell in Equation-23 can be approximated with the

$$\log \left[\int_{\mu_{cg}} \Pr\left[Y_{cg}, \mu_{cg}\right] d\mu_{cg} \right] \approx \frac{1}{2} \left[\log(2\pi) - \log\left(h\left[\hat{\mu}_{cg}\right]\right)\right] \tag{24}$$

where $\hat{\mu}_{cg}$ is the maximizer of $\Pr\left[Y_{cg}, \mu_{cg}\right]$ over $\mu_{cg}$and $h\left[\mu_{cg}\right]$ is the second derivative of $\Pr\left[Y_{cg}, \mu_{cg}\right]$ over μ_{cg}.

In order to assess the performance of the Laplace's method, we have compared the $\widehat{\beta}_1$, the estimated coefficient associated with the group indicator in the two group comparison settings in the Zeisel et al. data using Laplace's method (Laplace) and adaptive quadrature (Integration) in Figure S30. The estimates are highly correlated, indicating Laplace's method can give accurate estimates for the parameters of interests, under a wide range of sample sizes.

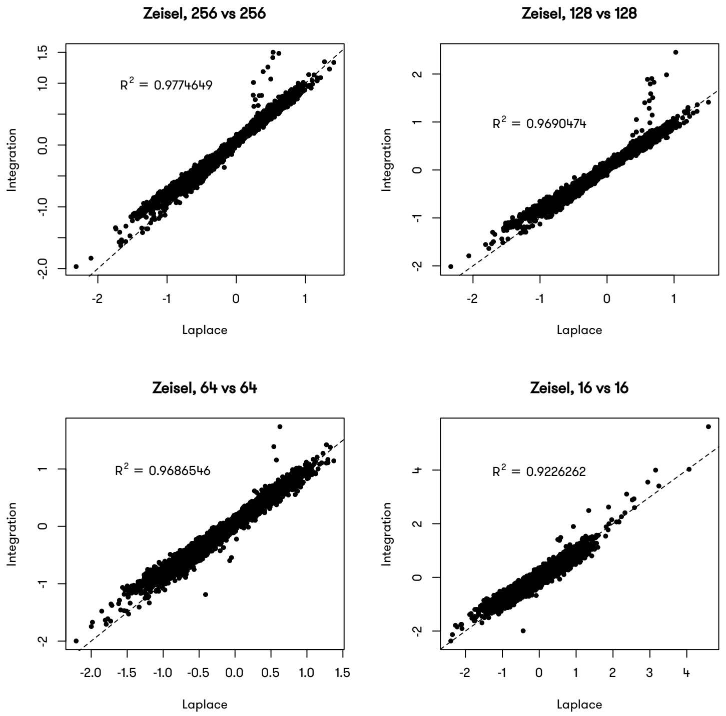

Figure S30: Comparison of Laplacian approximation and Adaptive Integration

Using Laplace's method can greatly reduce the CPU time required, as is show in Figure S31.

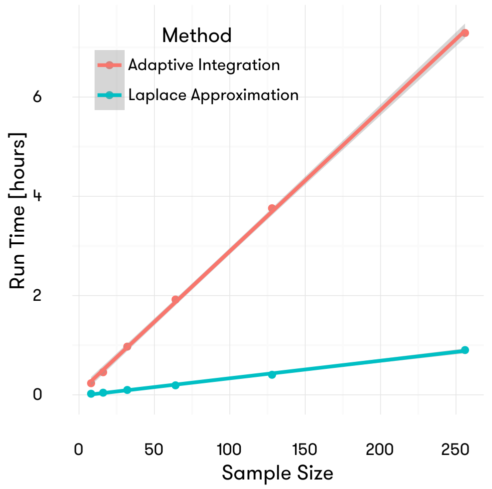

Figure S31: Comparison of run time of Laplacian approximation and Adaptive Integration, using 24 cores.

3.2 Session info for running DESeq2, MAST, SCDE

```
R version 3.2.2 (2015-08-14)
Platform: x86_64-redhat-linux-gnu (64-bit)
Running under: Red Hat Enterprise Linux Server release 6.6 (Santiago)
```

```
locale:
[1] LC_CTYPE=en_US.UTF-8       LC_NUMERIC=C
[3] LC_TIME=en_US.UTF-8        LC_COLLATE=en_US.UTF-8
[5] LC_MONETARY=en_US.UTF-8    LC_MESSAGES=en_US.UTF-8
[7] LC_PAPER=en_US.UTF-8       LC_NAME=C
[9] LC_ADDRESS=C               LC_TELEPHONE=C
[11] LC_MEASUREMENT=en_US.UTF-8 LC_IDENTIFICATION=C

attached base packages:
[1] parallel  stats4    stats     graphics  grDevices utils     datasets
[8] methods   base

other attached packages:
[1] MAST_0.931                 reshape_0.8.6
[3] scde_1.99.0                flexmix_2.3-13
[5] lattice_0.20-34            DESeq2_1.10.1
[7] RcppArmadillo_0.7.600.1.0  Rcpp_0.12.8
[9] SummarizedExperiment_1.0.2 Biobase_2.30.0
[11] GenomicRanges_1.22.4       GenomeInfoDb_1.6.3
[13] IRanges_2.4.8              S4Vectors_0.8.11
[15] BiocGenerics_0.16.1

loaded via a namespace (and not attached):
[1] edgeR_3.12.1         splines_3.2.2        Formula_1.2-1
[4] assertthat_0.1       latticeExtra_0.6-28  RSQLite_1.1-1
[7] quantreg_5.29        limma_3.26.9         digest_0.6.10
[10] RColorBrewer_1.1-2   XVector_0.10.0       minqa_1.2.4
[13] colorspace_1.3-2     htmltools_0.3.5      Matrix_1.2-7.1
[16] plyr_1.8.4           XML_3.98-1.5         SparseM_1.74
[19] genefilter_1.52.1    zlibbioc_1.16.0      xtable_1.8-2
[22] scales_0.4.1         brew_1.0-6           lme4_1.1-12
[25] BiocParallel_1.4.3   MatrixModels_0.4-1   htmlTable_1.7
[28] tibble_1.2           openssl_0.9.5        annotate_1.48.0
[31] mgcv_1.8-16          car_2.1-4            ggplot2_2.2.0
[34] Lmoments_1.2-3       RMTstat_0.3          nnet_7.3-12
[37] lazyeval_0.2.0       pbkrtest_0.4-4       survival_2.40-1
[40] magrittr_1.5         distillery_1.0-2     memoise_1.0.0
[43] nlme_3.1-128         MASS_7.3-45          foreign_0.8-67
[46] Cairo_1.5-9          Rook_1.1-1           tools_3.2.2
[49] data.table_1.10.0    extRemes_2.0-8       stringr_1.1.0
[52] munsell_0.4.3        locfit_1.5-9.1       cluster_2.0.5
[55] AnnotationDbi_1.32.3 lambda.r_1.1.9       base64_2.0
[58] pcaMethods_1.60.0    nloptr_1.0.4         futile.logger_1.4.3
[61] grid_3.2.2           rjson_0.2.15         gtable_0.2.0
[64] abind_1.4-5          DBI_0.5-1            gridExtra_2.2.1
[67] knitr_1.15.1         Hmisc_4.0-1          futile.options_1.0.0
[70] modeltools_0.2-21    stringi_1.1.2        geneplotter_1.48.0
```

```
[73] rpart_4.1-10         acepack_1.4.1
```

3.3 Session info for running SCRAN

```
R version 3.3.1 (2016-06-21)
Platform: x86_64-redhat-linux-gnu (64-bit)
Running under: Red Hat Enterprise Linux Server release 6.6 (Santiago)

locale:
[1] LC_CTYPE=en_US.UTF-8       LC_NUMERIC=C
[3] LC_TIME=en_US.UTF-8        LC_COLLATE=en_US.UTF-8
[5] LC_MONETARY=en_US.UTF-8    LC_MESSAGES=en_US.UTF-8
[7] LC_PAPER=en_US.UTF-8       LC_NAME=C
[9] LC_ADDRESS=C               LC_TELEPHONE=C
[11] LC_MEASUREMENT=en_US.UTF-8 LC_IDENTIFICATION=C

attached base packages:
[1] parallel  stats     graphics  grDevices utils     datasets  methods
[8] base

other attached packages:
[1] scran_1.0.4         scater_1.0.4        ggplot2_2.1.0
[4] Biobase_2.32.0      BiocGenerics_0.18.0 BiocParallel_1.6.6

loaded via a namespace (and not attached):
[1] Rcpp_0.12.7           plyr_1.8.4            zlibbioc_1.18.0
[4] bitops_1.0-6          viridis_0.3.4         tools_3.3.1
[7] biomaRt_2.28.0        digest_0.6.10         lattice_0.20-34
[10] rhdf5_2.16.0          RSQLite_1.0.0         tibble_1.2
[13] gtable_0.2.0          Matrix_1.2-7.1        shiny_0.14.2
[16] DBI_0.5-1             gridExtra_2.2.1       stringr_1.1.0
[19] dplyr_0.5.0           S4Vectors_0.10.3      IRanges_2.6.1
[22] stats4_3.3.1          grid_3.3.1            shinydashboard_0.5.3
[25] data.table_1.9.6      R6_2.2.0              AnnotationDbi_1.34.4
[28] XML_3.98-1.4          limma_3.28.21         reshape2_1.4.2
[31] magrittr_1.5          edgeR_3.14.0          matrixStats_0.51.0
[34] scales_0.4.0          htmltools_0.3.5       dynamicTreeCut_1.63-1
[37] tximport_1.0.3        assertthat_0.1        mime_0.5
[40] xtable_1.8-2          colorspace_1.2-7      httpuv_1.3.3
[43] stringi_1.1.2         RCurl_1.95-4.8        munsell_0.4.3
[46] rjson_0.2.15          chron_2.3-47          zoo_1.7-13
```

3.4 Code snippets for running DESeq2, MAST, SCDE and SCRAN

DESeq2:

```
library(DESeq2)
colData <- data.frame(conditions = x)
rownames(colData) <- colnames(y_data)
dds <- DESeqDataSetFromMatrix(countData = y_data, colData = colData, design
```

```
featureData <- data.frame(gene=rownames(y_data))
mcols(dds) <-DataFrame(mcols(dds), featureData)

library(BiocParallel)
register(MulticoreParam(n.cores))
dds_est <- DESeq(dds, parallel = TRUE)
res <- results(dds_est, parallel = TRUE)
```

MAST:

```
library(MAST)
library(reshape)
y_data$Gene <- rownames(y_data)
y_data_melted <- melt(y_data, id.vars = c('Gene'))
y_data_melted$condition <- rep(x, each=(dim(y_data)[[1]]))
colnames(y_data_melted)[2]<-'Cell'
y_data_sca <- SingleCellAssay(dataframe = y_data_melted, idvars = c('Cell','
zlm.output <- zlm.SingleCellAssay(~condition, y_data_sca, method='glm',ebaye
zlm.srt <- lrTest(zlm.output,'condition')
```

SCDE:

```
sg<-factor(paste('GROUP_',x,sep=''), levels=c('GROUP_0','GROUP_1'))
names(sg) <- colnames(y_data)
cd <- y_data
# omit genes that are never detected
cd <- cd[rowSums(cd)>0, ]
cd <- apply(cd, 2, function(x) {storage.mode(x) <- 'integer';x})
library(scde)
o.ifm <- scde.error.models(counts = cd, groups = sg, n.cores = n.cores, thre
valid.cells <- o.ifm$corr.a > 0
print(valid.cells)
print(table(valid.cells))
o.ifm <- o.ifm[valid.cells, ]
o.prior <- scde.expression.prior(models = o.ifm, counts = cd, length.out =
# run differential expression tests on all genes.
ediff <- scde.expression.difference(o.ifm, cd, o.prior, groups = sg, n.rando
```

SCRAN:

```
if(!isErccEnabled){
sce <- newSCESet(countData=data.frame(y))
isSpike(sce) <- rep(c(FALSE), dim(y)[[1]])

sce <- computeSumFactors(sce)
} else {
sce <- newSCESet(countData=data.frame(rbind(y,ercc)))
MySpikes <- rep(c(FALSE, TRUE), c(dim(y)[[1]], dim(ercc)[[1]]))
isSpike(sce) <- MySpikes
sce <- computeSpikeFactors(sce)
}
```

```
sce <- normalize(sce)


x <- t(x)[,-1]
design <- model.matrix(~x)
if (!isDESeq) {
edger.y <- convertTo(sce, type="edgeR")
library(edgeR)
y <- estimateDisp(edger.y, design, prior.df=0, trend='none')
fit <- glmFit(y, design)
res <- glmTreat(fit, lfc=1)
save('design','edger.y','res','fit', file = output.filename)
} else {
library(DESeq2)
deseq.y <- convertTo(sce, type='DESeq2')
red.design <- model.matrix(~1)

register(MulticoreParam(n.cores))
dd_est <- DESeq(deseq.y, full = design, parallel = TRUE)
res <- results(dd_est, parallel = TRUE)
save('design','deseq.y','res', file = output.filename)
}
```

References

1. Zeisel, A. *et al.* Cell types in the mouse cortex and hippocampus revealed by single-cell RNA-seq. *Science* **347,** 1138–1142 (2015).
2. Wills, Q. F. *et al.* Single-cell gene expression analysis reveals genetic associations masked in whole-tissue experiments. *Nat Biotech* **31,** 748–752 (2013).
3. Bengtsson, M., Stahlberg, A., Rorsman, P. & Kubista, M. Gene expression profiling in single cells from the pancreatic islets of Langerhans reveals lognormal distribution of mRNA levels. *Genome Res* **15,** 1388–1392 (2005).
4. Kharchenko, P. V., Silberstein, L. & Scadden, D. T. Bayesian approach to single-cell differential expression analysis. *Nat Meth* **11,** 740–742 (2014).
5. Finak, G. *et al.* MAST: a flexible statistical framework for assessing transcriptional changes and characterizing heterogeneity in single-cell RNA sequencing data. *Genome Biology* **16,** 1–13 (2015).
6. Love, M. I., Huber, W. & Anders, S. Moderated estimation of fold change and dispersion for RNA-seq data with DESeq2. *Genome Biol* **15,** 550 (2014).
7. Lun, A. T. L., Bach, K. & Marioni, J. C. Pooling across cells to normalize single-cell RNA sequencing data with many zero counts. *Genome Biology* **17,** 75 (2016).
